# The lncRNA LINC01605 promotes the progression of pancreatic ductal adenocarcinoma by activating the mTOR signaling pathway

**DOI:** 10.1186/s12935-024-03440-z

**Published:** 2024-07-24

**Authors:** Yu-Heng Zhu, Qin-Yuan Jia, Hong-Fei Yao, Zong-Hao Duan, Xue-Shi-Yu Ma, Jia-Hao Zheng, Yi-Fan Yin, Wei Liu, Jun-Feng Zhang, Rong Hua, Ding Ma, Yong-Wei Sun, Jian-Yu Yang, De-Jun Liu, Yan-Miao Huo

**Affiliations:** 1 Department of Biliary-Pancreatic Surgery, Renji Hospital, School of Medicine, Shanghai Jiao Tong University, Shanghai, 200127 China; 2 Department of Hepato-Biliary-Pancreatic Surgery, General Surgery, Huadong Hospital Affiliated to Fudan University, Shanghai, 200040 China; 3 State Key Laboratory of Systems Medicine for Cancer, Shanghai Cancer Institute, Renji Hospital, School of Medicine, Shanghai Jiao Tong University, Shanghai, 200240 China

**Keywords:** Pancreatic ductal adenocarcinoma, Long intergenic non-protein coding RNA 1605, Liver metastasis, mTOR signaling pathway, Cholesterol metabolism

## Abstract

**Background:**

This study investigated the molecular mechanism of long intergenic non-protein coding RNA 1605 (LINC01605) in the process of tumor growth and liver metastasis of pancreatic ductal adenocarcinoma (PDAC).

**Methods:**

LINC01605 was filtered out with specificity through TCGA datasets (related to DFS) and our RNA-sequencing data of PDAC tissue samples from Renji Hospital. The expression level and clinical relevance of LINC01605 were then verified in clinical cohorts and samples by immunohistochemical staining assay and survival analysis. Loss- and gain-of-function experiments were performed to estimate the regulatory effects of LINC01605 in vitro. RNA-seq of LINC01605-knockdown PDAC cells and subsequent inhibitor-based cellular function, western blotting, immunofluorescence and rescue experiments were conducted to explore the mechanisms by which LINC01605 regulates the behaviors of PDAC tumor cells. Subcutaneous xenograft models and intrasplenic liver metastasis models were employed to study its role in PDAC tumor growth and liver metastasis in vivo.

**Results:**

LINC01605 expression is upregulated in both PDAC primary tumor and liver metastasis tissues and correlates with poor clinical prognosis. Loss and gain of function experiments in cells demonstrated that LINC01605 promotes the proliferation and migration of PDAC cells in vitro. In subsequent verification experiments, we found that LINC01605 contributes to PDAC progression through cholesterol metabolism regulation in a LIN28B-interacting manner by activating the mTOR signaling pathway. Furthermore, the animal models showed that LINC01605 facilitates the proliferation and metastatic invasion of PDAC cells in vivo.

**Conclusions:**

Our results indicate that the upregulated lncRNA LINC01605 promotes PDAC tumor cell proliferation and migration by regulating cholesterol metabolism via activation of the mTOR signaling pathway in a LIN28B-interacting manner. These findings provide new insight into the role of LINC01605 in PDAC tumor growth and liver metastasis as well as its value for clinical approaches as a metabolic therapeutic target in PDAC.

**Supplementary Information:**

The online version contains supplementary material available at 10.1186/s12935-024-03440-z.

## Background

Pancreatic ductal adenocarcinoma (PDAC) is widely recognized as a highly malignant tumor with a staggeringly high mortality rate. According to the cancer statistics of the American Cancer Society, PDAC ranks among the leading ten tumors in terms of incidence and ranks 3rd in terms of the number of cancer-related deaths; it has the lowest 5-year survival rate (11%) among the assessed cancer types [1]. Despite advancements in chemotherapy, targeted therapy, radiotherapy and immunotherapy, surgical resection remains the most effective treatment for PDAC [2, 3]. Disappointingly, only 15–20% of diagnosed PDAC patients have the chance to undergo surgery at diagnosis, primarily due to distant metastasis, which is the manifestation of high malignant potential. Distant metastasis is observed in over 50% of cases at the time of initial diagnosis, with the liver being the most preferred site of metastasis [3, 4]. In addition to its hindering effect in treatment, liver metastasis contributes to poor patient prognosis since it is related to recurrence and systemic disorders. Therefore, the mechanisms of local progression and liver metastasis in PDAC have long been the major focus in the field of PDAC; their further study has scientific significance and research value, as it may reveal potential therapeutic targets and new treatment strategies.

Research in recent years has provided increasing evidence that lncRNAs are capable to regulate the genesis and development of tumors since they can bind to DNA, RNA, or even proteins to control the levels of gene expression [5–7]. Long intergenic non-protein coding RNA 1605 (LINC01605) is located on chromosome 8p11.23 with a length of 1663 bp. The function of LINC01605 has been reported in colorectal cancer, bladder cancer, breast cancer, nasopharyngeal carcinoma and squamous cell carcinoma [8–13]. However, there have been no reports on one of the most malignant gastrointestinal tumors, PDAC. Therefore, we aimed to explore the potential mechanisms of LINC01605 in PDAC progression to further understand the role of LINC01605 and to find potential targets for PDAC.

The mammalian/mechanist target of rapamycin (mTOR) signaling pathway mainly regulates cell growth and cell metabolism. Between the two branches of the signaling pathway, mTOC1 signaling can regulate cell metabolism by phosphorylating and activating p70 S6 kinase (S6K), while the effect of mTORC2 signaling is less delineated [14, 15]. Unsurprisingly, mTOR signaling is frequently hyperactivated in cancer, which contributes to the nutrient metabolism reprogramming of cancer cells. Notably, the mTOR-mediated signaling pathway, especially mTORC1 signaling, has the ability to facilitate tumor cell survival, migration and metastasis through the P70 S6K-mediated pathway as well as the 4EBP1-mediated pathway [14, 16–19]. Therefore, we aimed to assess whether there is a lncRNA that participates in the regulation of the mTOR signaling pathway.

This study embarked on elucidating the role of lncRNAs in PDAC progression. We filtered out and focused on LINC01605 and verified its expression pattern and prognostic value with our clinical samples and PDAC cell lines. Further experiments demonstrated its ability to regulate cellular functions, including proliferation and migration in vitro and cell growth and metastatic invasion in vivo. Importantly, we found that LINC01605 promotes PDAC progression through mTOR signaling pathway activation, which further regulates cholesterol metabolism and thus leads to PDAC tumor growth and liver metastasis; we also found that it may interact with LIN28B. Therefore, our study proposes a possible lncRNA marker for predicting prognosis and a potential therapeutic target for inhibiting PDAC progression.

## Methods

### Clinical samples of patients and tissue microarray

The clinical samples of PDAC primary tumors, PDAC liver metastasis, and the Tumor Micro Array (TMA) included in this study were obtained from Renji Hospital, School of Medicine, Shanghai Jiao Tong University (RA-2019-116, approved by the Research Ethics Committee of Ren Ji Hospital, School of Medicine, Shanghai Jiao Tong University). Among them, the TMA contains a number of 130 pairs of PDAC primary tumor and the corresponding tumor-adjacent tissues as Supplementary Table 1 shows. The clinical samples were surgically obtained from PDAC patients and were confirmed by the postoperative pathology at our institution of Renji Hospital. Notably, the patients had not received any form of radiotherapy or chemotherapy before surgical resection. The survival time of each patient was calculated as the duration started from surgery to the definite date of PDAC-related death or the last possible date of follow-up. This study has undergone approval by the Research Ethics Committee of Renji Hospital, School of Medicine, Shanghai Jiao Tong University.

### Histology and immunohistochemistry (IHC) assays

All the patients involved in this study had been supplied with a printed informed consent form before our approved enrollment. The process of hematoxylin and eosin (H&E) staining assay as well as the immunohistochemistry (IHC) staining assay was performed according to the steps of our previous work [20].

The process of scoring was verified by two licensed pathologists independently. The score was then estimated based on the percentage of area with positive staining, where point 0 for 0%–5%, 1 for 6%–35%, 2 for 36%–70%, 3 for over 70%, and staining intensity, where point 0 stood for non-staining, point 1 stood for weakly staining, point 2 stood for moderately staining, and point 3 stood for strongly staining. Then the final score of each sample was comprehensively calculated as the percentage of positive staining score × the intensity of staining score and displayed as + for a score of 0–1, + for 2–3, ++ for 4–6, and +++ for over 6. For group dividing, the group of LINC01605 low expression was defined as those with a total score < 4, while the group of LINC01605 high expression involved those with a total score ≥ 4.

A specific probe detecting LINC01605 was designed and applied in this assay and the following experiments (Sequence in Supplement Table 1).The primary antibodies used in this assay included anti-Ki-67 (1:1000, #ab15580 Abcam), anti-Phospho-mTOR (Ser2481) (1:100, #2974, Cell Signaling Technology), anti-Phospho-p70 S6 Kinase (Thr421/Ser424) (1:100, #9204, Cell Signaling Technology), and anti-LIN28B (1:100, #16178-1-AP, Proteintech).

### Immunofluorescence (IF) assays

The cells for detection were seeded onto and cultured for 48 h on cell climbing sheets placed in 6-well plates. Next, the sheets underwent a 15-min 4% paraformaldehyde (#G1101, Servicebio) fix followed by a 10-min 0.1% Triton X-100 at room temperature before being taken out from the wells. Then the sheets were blocked in a 5% BSA solution for 1 h before incubation with the LINC01605 probe or specific primary antibodies like CK19 (1:200, #10712-1-AP, Proteintech), SREBP2 (1:200, # 28212-1-AP, Proteintech), Filipin III (#SAE0087, Sigma-Aldrich) and others mentioned above with the same dilution ratio at the 4 °C refrigerator overnight. On the next day, a 1-h incubation with the corresponding secondary antibodies was conducted at room temperature, during which the antibodies involved the FITC conjugated Goat Anti-Rabbit IgG (1:100, #GB22303, Servicebio), the Cy3 conjugated Goat Anti-Rabbit IgG (1:100, #GB21303, Servicebio), and the Cy5 conjugated Goat Anti-Rabbit IgG (1:100, # GB27303, Servicebio). A 5-min DAPI (#G1012, Servicebio) staining was performed to stain the nucleus.

For tissues on sections, the paraffin sections were dewaxed and fixed by citrate. Then the tissue samples on the sections were fully covered and incubated with specific primary antibodies (same as above) overnight at the 4 °C refrigerator. After the phosphate buffer solution (PBS, #G2156, Servicebio) washing, the tissue samples were then incubated with the secondary antibodies protected from light exposure for 1 h at room temperature. A 5-min DAPI was used to stain the nucleus.

Confocal microscopes (Leica, Germany) were employed to read and snap the images.

### Cell culture and reagents

The human PDAC cell lines including ASPC-1, BXPC-3, CFPAC, MiaPaca-2, PANC-1, Patu-8988 and SW-1990 used in this study were all preserved in the Shanghai Cancer Institute, RenJi Hospital, School of Medicine, Shanghai Jiao Tong University. All the cells used were strictly cultured in the recommended medium according to the American Type Culture Collection (ATCC), with a 10% (v/v) fetal bovine serum (FBS) and a 1% streptomycin/penicillin (P/S) antibiotics in a humidification-kept incubator under the condition of 5% CO_2_ and 37 °C in the HERAcell 150i CO_2_ incubator (Thermo Scientific, USA).

Dimethyl sulfoxide (DMSO, # HY-Y0320) was purchased from MedChemExpress. Puromycin (#60209ES) was purchased from YEASEN Biotech. Rapamycin (#HY-10219, MedChemExpress) was used strictly in accordance with our previous work as 10 nM for cell function experiments and 50 nM 24–48 h for mRNA or protein level detection [20].

### Quantitative real-time polymerase chain reaction (qRT-PCR) analysis

The RNAiso Plus (#9109, Takara Bio) was employed in the extraction of PDAC cells total RNA as the manufacturer’s protocols indicated. The PrimeScriptTM RT Reagent Kit (#RR037A, Takara Bio) was used in the process of reverse transcription from RNA into cDNA.

The qRT-PCR assay was carried out with the TB Green Premix Ex TaqTM II (#RR820A, Takara Bio) on the Applied Biosystems™ 7500 Real-Time PCR Systems (Thermo Scientific, USA) according to the recommended thermal cycle settings (a 10-min initial cycle at 95.0 °C, 40 cycles of 10 s from 95.0 °C to 30 s 60.0 °C). The relative mRNA expression was respectively normalized to the 18 s RNA level. Supplementary Table 2 lists the sequences of the primer used in amplifying the target genes.

### Western blotting (WB) analysis

The RIPA Lysis and Extraction Buffer (#89900, ThermoFisher) was used in the extraction of the PDAC cells total protein. The protein samples were next quantified by the bicinchoninic acid (BCA) Protein Assay Kit (#SB-WB013, ShareBio). An equal amount of the proteins within the lysates were then separated by the sodium dodecyl sulfate–polyacrylamide gel electrophoresis (SDS-PAGE) and then rapidly transferred onto the nitrocellulose membranes. After that, the membranes with protein bands were blocked within a 5% skim milk, cut, and incubated separately with target-specific primary antibodies relatively at 4 °C at 60 rpm overnight. The next day, the membranes were correspondingly incubated with species-specific secondary antibodies for an hour at room temperature. The immunoreactions were detected by a ChemiDoc XRS + chemiluminescence fluorescence imaging system (#1708265, BIO-RAD).

The primary antibodies involved in our study included anti-mTOR (1:1000, #2983, Cell Signaling Technology), anti-Phospho-mTOR (Ser2481) (1:1000, #2974, Cell Signaling Technology), anti-p70 S6 Kinase (1:1000, #9202, Cell Signaling Technology), anti-Phospho-p70 S6 Kinase (Thr421/Ser424) (1:1000, #9204, Cell Signaling Technology), anti-LIN28B (1:1000, #16178-1-AP, Proteintech), and anti-β-actin (1:2000, #4970, Cell Signaling Technology).

The only secondary antibody in this assay was the goat anti-rabbit IgG, HRP-linked antibody (1:5000, #7074, Cell Signaling Technology).

### Gene knockdown and overexpression assays

The lentiviruses carrying scramble sequences for Negative Control (NC) and short hairpin RNA (shRNA) sequences used for transfection with shRNA were obtained from OBiO Technology (Shanghai) Co., Ltd., sequences of which are listed in Supplement Table 3.

Small interfering RNA (siRNA) of NC, LINC01605 and Lin28b were obtained from Tsingke Biotech Co., Ltd. (Beijing), sequences of which are listed in Supplement Table 3.

The pSLenti-pA-MCS-CMV-EF1-Puro-WPRE lentivirus was employed as control (Vector, Vec in following), and the pSLenti-pA-LINC01605-CMV-EF1-Puro-WPRE lentivirus was employed for LINC01605 overexpression.

The lentiviruses were transfected using a 1:200 polybrene (OBiO Technology) according to instructions and the cells were screened by a 5 μg/mL puromycin.

The siRNAs were transfected using the jetPRIME transfection reagent (Polyplus, France) according to the instructions of the manufacturer.

### Cell proliferation assays

The Cell Counting Kit-8 (CCK-8) (#SB-CCK8, Share-Bio) assay was employed for cell proliferation estimation. An initial number of 1500 PDAC cells were resuspended and then seeded into the 96-well plates with quintuple duplicates. Starting from the moment of cell adherence, measurements were taken every 24 h for 4–5 days. Before the measurement, the cell culture medium was replaced by a 10% CCK-8 medium was added to each well and incubated for 2 h at 37 °C. The absorbance values at 450 nm were measured by the automatic enzyme-linked immune detector (#M1000 PRO, Tecan, Switzerland), and the data was then collected and plotted as growth curves for comparative analysis. The CCK-8 assay in our study was independently repeated three times.

Another assay detecting cell proliferation performed was the colony formation assay. For each group, an initial number of 1500 PDAC cells were resuspended and evenly seeded into 6-well plates for each well with triplicates and cultured in the incubator for 14 days. Afterward, the colonies formed were washed with PBS, fixed using a 4% paraformaldehyde for 15 min and then stained with a 0.1% crystal violet (#G1014, Servicebio) for another 15 min. Then, Photographs were scanned and the colonies were counted by area. Three independent experiments were performed as well.

### Cell migration and invasion assays

For the Transwell assay, an amount of 5 × 10^4^ resuspended PDAC cells were evenly planted into the 6.5 mm Transwell chamber with 8 μm pores (Costar, Corning Incorporated, USA) placed upon the wells of the 24-well plate. For each chamber within a well, there was a volume of 300 μL serum-free medium with cells inside the chamber and a volume of 700 μL cell culture medium with 10% FBS outside. After 36 h at 37 °C, the chambers with PDAC cells on the membranes were harvested, PBS washed and 4% paraformaldehyde-fixed for 15 min, and underwent a 15-min 0.1% crystal violet staining for further observation. At least three random fields were counted at a 100 × zoom under the inverted fluorescence microscope (Zeiss AXIOVERT 200, Germany) for each group in triplicate.

For the wound healing assay, the treated PDAC cells with a density of about 100% cultured in 6-well plates were straightly scratched by a sterilized tip to create a wound. Then the plates with PDAC cells were observed and photographed for further measurement by the time of 0 h (immediately after scratching) and after 24 h with the inverted fluorescence microscope (Zeiss AXIOVERT 200, Germany) at a 100 × zoom for at least three random fields for each group in triplicate.

### RNA sequencing (RNA-seq) analysis

The shNC and shLINC01605-1 transfected PDAC cells’ total RNA for sequencing was extracted as described above. Then the RNA quality was quantified, and then quality-approved by the 5300 Bioanalyser (Agilent Technologies). Next, the samples were further transferred to Xuran Biological Co., Ltd. (Shanghai, China), which were processed according to the standard protocols there. Briefly, the fragments of the samples were first purified with the AMPure XP system (Beckman Coulter, Beverly, USA). After the process of cluster generation with the cBot Cluster Generation System by TruSeq PE Cluster Kit v3-cBot-HS (Illumia), the library sequencing was conducted by the Illumina Hiseq X Ten and the reads numbers were 150 bp-size-selectively counted by HTSeq v0.6.0. Last, the FPKM of each gene was calculated.

### Total cholesterol detection assays

The levels of the intracellular total cholesterol of the PDAC cells were detected by the Cholesterol/Cholesteryl Ester Quantitation Assay kits (#ab65359, Abcam) according to the protocols from the manufacturer. An amount of 1 × 10^6^ PDAC cells for each sample with triplicates was harvested and counted for measurement. The 96-well plates with samples incubated with the specific cholesterol probe were measured at the absorbance of 570 nm using the automatic enzyme-linked immune detector (#M1000 PRO, Tecan, Switzerland) for data collection and further analysis.

### Animal model studies

The animal experiments were strictly carried out under approval by Renji Hospital Animal Care and Use Committee (Shanghai, P.R. China). The mice included in our study were manipulated following the Guide for the Care and Use of Laboratory Animals prepared by the National Academy of Sciences and published by the NIH (Bethesda, MD).

For the subcutaneous xenograft mouse models, Wild-Type male nude mice of 8-week-old weighting from 20 to 25 g were used for subcutaneous implantation. A number of 2 × 10^6^ of either the Patu-8988 (transfected with shNegativeControl/shLINC01605-1) or the PANC1 (infected with Vector/LINC01065) resuspended in a 100 μL PBS was injected into one side back of each nude mouse. The volumes of the tumors were measured and calculated per week, and the weight was measured after 4 weeks when the tumors were resected. Tumor volumes were roughly calculated as v = 4 × (1/2tumor length)^3^. Then the tumors were fixed by the 4% paraformaldehyde and preserved for further experiments.

For the intrasplenic injection liver metastasis mouse models, Wild-Type male nude mice of 8-week-old weighting from 20 to 25 g were used for modeling. A number of 1 × 10^6^ of the same groups of Patu-8988 or PANC1 cells mentioned above, which was resuspended in a 25 μL PBS, was injected into the spleen followed by a 2 s needle retention for leakage-prevention and a rapid wound closure to complete the modeling process. After 5 weeks (for the Patu-8988 group, and an early harvest at 2 weeks for the PANC1 group), the intrasplenic injection model mice were sacrificed for liver harvesting in detection of possible liver metastasis. Then, the livers of the mice were fixed in 4% paraformaldehyde and preserved for further experiments. Additionally, an extra paired group of mice for each experiment was set for a survival time observation with a termination of 5 weeks. Detailed methods can be searched up in our previous work [21].

### Statistical analysis

The processing and analysis of the data in our study were performed by ImageJ (version 2.1.0), GraphPad Prism (version 8.0), R (version 4.0.2) and SPSS (version 22.0). Specifically, ImageJ was used for cell counting and quantification. GraphPad Prism was employed for statistical analyses and graphical representation of differences in LINC01605 expression levels between tumor and normal tissues in the database, TMA categorization, mRNA expression of LINC01605 in PDAC cell lines, outcomes from CCK-8, colony formation, Transwell and wound healing assays, as well as the quantification and visualization of cellular total cholesterol. The R was utilized for screening and intersecting differentially expressed genes from public databases such as TCGA and GSE. SPSS was applied for organizing and analyzing the TMA cohort dataset.

Data in this study were finally presented as the means ± standard error of the mean (SEM). The P value stood for the statistical significance of differences between the data groups, and P value < 0.05 was taken as a standard for statistical significance. Continuous variables in this study were presented as mean ± standard error of the mean (SEM), and comparisons between groups were performed using independent samples Student's t-test or one-way analysis of variance (ANOVA). Relationships between continuous variables were assessed using Pearson correlation coefficient or Spearman rank correlation test, whereas categorical variables were evaluated using chi-square tests. In the survival analysis, the association between variables and overall survival rates was investigated using the Kaplan–Meier method, with log-rank tests used to analyze differences in survival curves. Univariate and multivariate analyses were carried out using the Cox proportional hazards regression model. In this study, ns stood for p > 0.05; and *, p < 0.05; **, p < 0.01; ***, p < 0.001.

## Results

### Upregulated LINC01605 expression is related to tumor progression and an unfavorable prognosis in PDAC

**Fig. 1:**
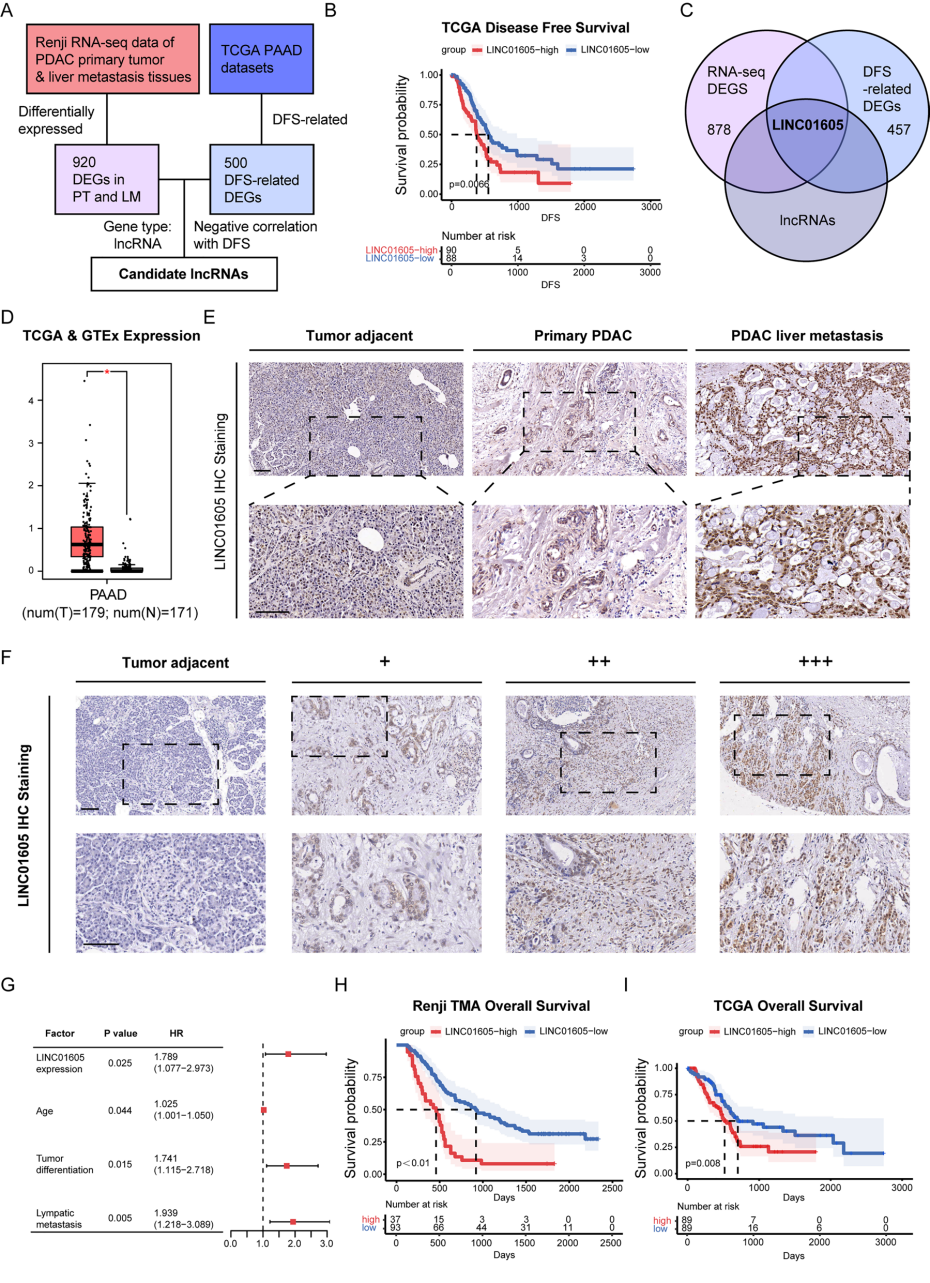
LINC01605 expression is abnormally up-regulated in PDAC primary tumor and liver metastasis tissues and is of prognostic value. **A** The sketch of filtering out LINC01605 as a positive factor of PDAC liver metastasis. DEG: differentially expressed genes; DFS: disease-free survival; PT: primary tumor; LM: liver metastasis. **B** The Kaplan–Meier analysis on DFS of the TCGA PAAD datasets grouped by LINC01605 expression. p = 0.0066. **C** LINC01605 is filtered out by the Intersection of the 920 DEGs from RNA-seq and the top 500 TCGA DFS-related genes focusing on lncRNA. **D** The expression of LINC01605 is relatively up-regulated according to the TCGA and GTEx datasets. **E** LINC01605 is relatively highly expressed in PDAC primary tumor and liver metastasis tissues than in the tumor-adjacent ones by the IHC staining of samples from the Renji Hospital. Scale bar, 100 μm. **F** Standard IHC staining images of LINC01605 expression in the Renji TMA for PDAC primary tumor tissues representing each score and corresponding adjacent tissues. Scale bar, 100 μm. **G** Multivariate Cox regression analysis on overall survival of the Renji TMA cohort by the clinical/pathological factors. HR: hazard ratio. **H**, **I** The Kaplan–Meier analysis on overall survival of the Renji TMA cohort (**H**, p < 0.01) and TCGA PAAD database (**I**, p = 0.008) based on LINC01605 expression

To explore whether lncRNAs function as critical factors in PDAC tumor growth and liver metastasis, we combined two sets of differentially expressed genes (DEGs) to identify possible candidates (Fig. 1A). One was the RNA-seq dataset of the paired PDAC primary tumor and liver metastasis tissues with adjacent normal tissues by previous work of our own institution [22], while the other was the top 500 DEGs analyzed from The Cancer Genome Atlas (TCGA) PAAD datasets. Interestingly, by focusing on lncRNAs, we found that LINC01605 was the only lncRNA correlated with DFS (Fig. 1B) within the intersection of the two DEG sets (Fig. 1C). Additionally, the upregulation of LINC01605 expression in PDAC tumor tissues was further verified by expression analysis of the TCGA & Genotype-Tissue Expression (GTEx) PAAD datasets (Fig. 1D). Therefore, we targeted LINC01605 as a potential lncRNA regulating PDAC progression and conducted subsequent verification. First, to further investigate the expression pattern of LINC01605 in PDAC, we designed a specific molecular probe to detect LINC01605 and performed IHC staining on our clinical pathology-verified PDAC primary tumor and liver metastasis specimens as well as the corresponding tumor-adjacent tissues obtained from Renji Hospital, School of Medicine, Shanghai Jiao Tong University. LINC01605-specific staining demonstrated that the expression level was increased in both the primary tumor and the liver metastasis tissues compared to that in the adjacent tissues (Fig. 1E). To verify the expression and clinical relevance of LINC01605, we assessed a TMA containing 130 pathology-verified PDAC specimens with their matched adjacent tissues from our own institution for an IHC staining-based analysis. As shown in Fig. 1F, the tissue samples were analyzed, scored and grouped according to the LINC01605-positive area along with the staining intensity. As expected, we confirmed by multivariate Cox regression analysis that the LINC01605 expression level was significantly related to clinical or pathological factors such as age, tumor differentiation, and, importantly, lymph node metastasis (Fig. 1G). In addition, Kaplan‒Meier analysis also indicated that those patients with high LINC01605 expression had a relatively poor prognosis in terms of overall survival in our cohort (Fig. 1H) as well as in the TCGA PAAD cohorts (Fig. 1I). Altogether, these data and results suggest that LINC01605 may act as a key factor in the process of PDAC tumor growth and liver metastasis.

### LINC01605 promotes PDAC cell proliferation and migration in vitro

**Fig. 2:**
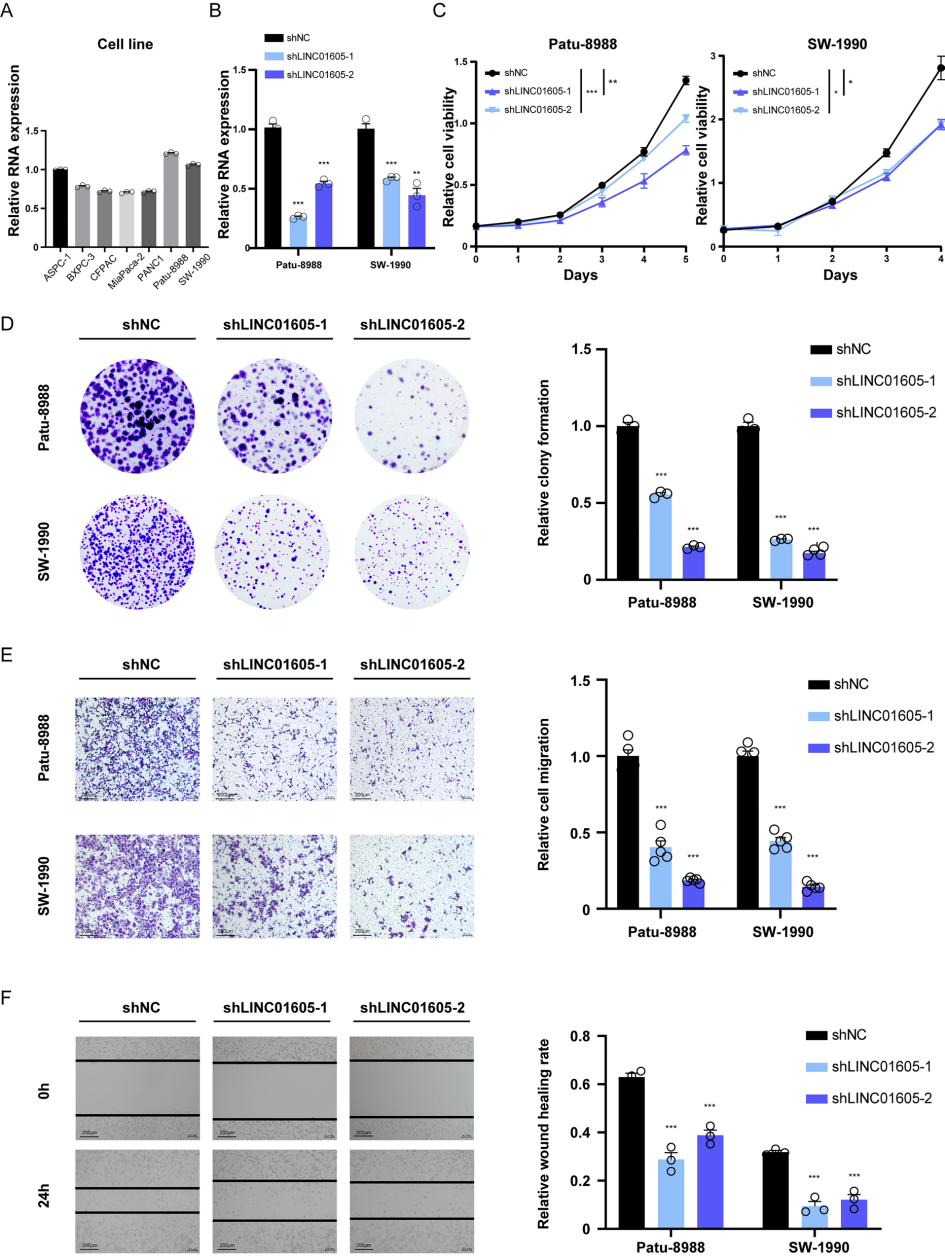
Inhibition of LINC01605 suppresses the cellular function of proliferation and migration of PDAC cells in vitro. **A** The relative RNA expression level of LINC01605 detected by qRT-PCR among 7 pancreatic cancer cell lines. **B** The relative LINC01605 expression level in LINC01605-knockdown (LINC01605-KD) pancreatic cancer cell lines Patu-8988 and SW-1990 by shRNA. **C** The CCK-8 assay of Patu-8988 and SW-1990 demonstrated that the cellular proliferation ability was suppressed by the effect brought by LINC01605 knockdown. **D** The colony-formation assay demonstrated that the LINC01605-KD Patu-8988 and SW-1990 had a lower ability of colony formation as a form of proliferation. **E** The Transwell assay demonstrated the restrained cellular migration ability of the LINC01605-KD Patu-8988 and SW-1990. Scale bar, 200. **F** The 24-h wound healing assay performed on the LINC01605-KD and control PDAC cells demonstrated the relatively lower migration ability of LINC01605-KD cells. Scale bar, 200 μm. (Patu-8988 shown as representative images)

**Fig. 3:**
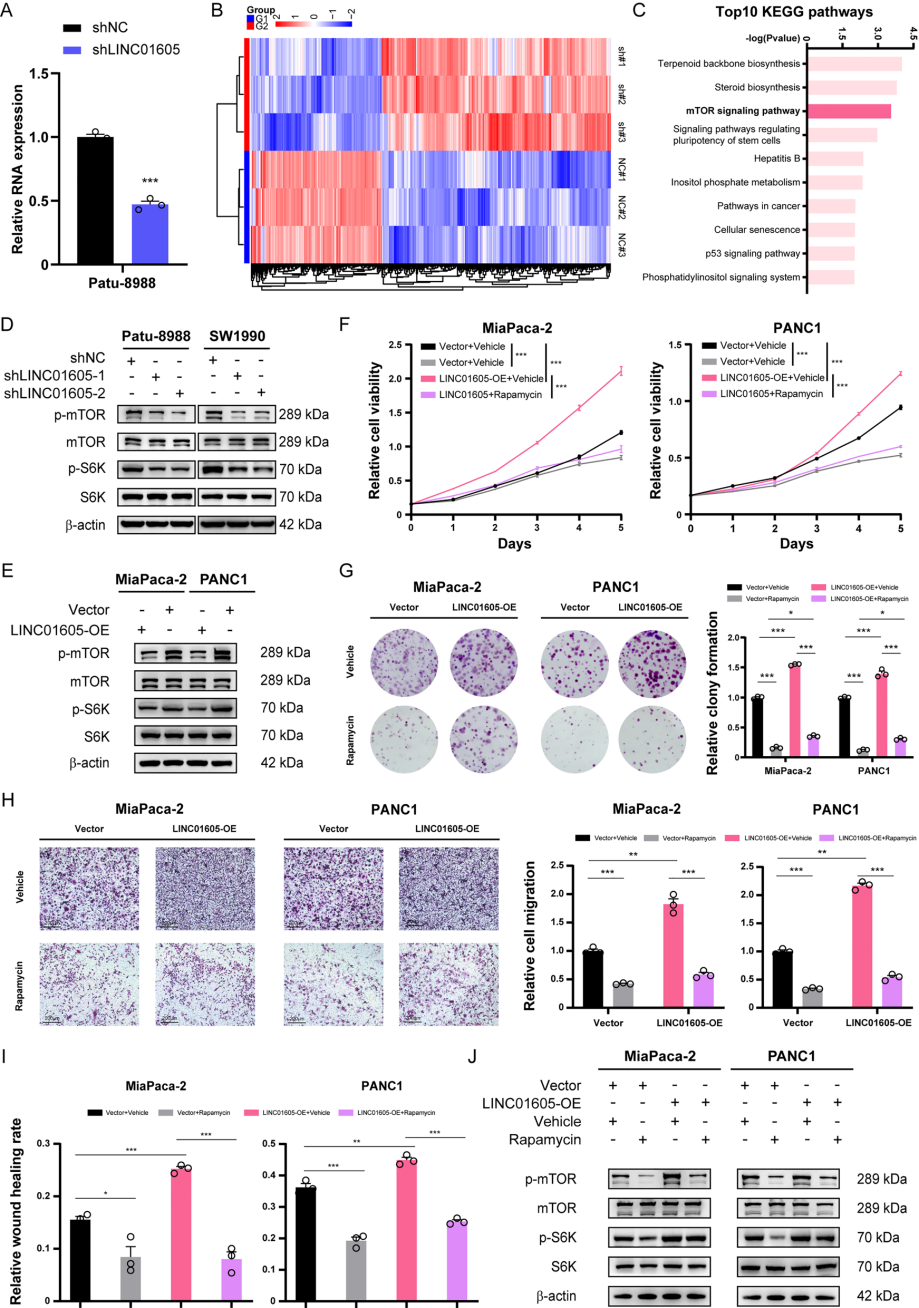
LINC01605 promoted PDAC cell proliferation and migration by activating the mTOR signaling pathway. **A** The relative LINC01605 expression level of the LINC01605-KD and LINC01605-NC Patu-8988 for RNA-seq. **B** The heatmap of the screened gene categories of the six samples clustered by bidirectional hierarchical clustering. **C** The Top 10 significantly enriched KEGG pathways ranked by −log(P value). **D**, **E** The protein expression level of phosphorylated and total mTOR, phosphorylated and total S6K and β-actin of the LINC01605-NC/LINC01605-KD PDAC cells (**D**) and Vector/LINC01605-OE PDAC cells (**E**) analyzed by Western blotting. **F** The CCK-8 assay demonstrated that the enhanced cellular proliferation ability of the two LINC01605-OE PDAC cell lines was partly blocked by the effect of Rapamycin (10 nM). (Vehicle, DMSO). **G** The colony formation assay represented the influence of Rapamycin (10 nM) on blocking the up-regulating effect of LINC01605 overexpression on cell proliferation. **H** The Transwell assay showed the blockage on the cellular migration ability of the LINC01605-OE and Vector cells by Rapamycin (10 nM). Scale bar, 200 μm. **I** The wound healing assay demonstrated a lower rate of migration of the Rapamycin-treated (10 nM) cells than the control ones by 24 h. **J** The protein expression level analyzed by Western blotting of phosphorylated and total mTOR, phosphorylated and total S6K and β-actin of the two Rapamycin-treated LINC01605-OE cell lines and the control groups (Rapamycin 50 nM, 36 h)

To investigate the regulatory function of LINC01605 on PDAC cells, we first conducted a qRT‒PCR assay to detect the RNA expression levels across seven commonly used PDAC cell lines: ASPC-1, BXPC-3, CFPAC, MiaPaca-2, PANC-1, Patu-8988 and SW-1990 (Fig. 2A). Following this, we silenced LINC01605 expression in Patu-8988 and SW-1990 cells and confirmed knockdown by qRT-PCR (Fig. 2B). We then assessed the effect of LINC01605 silencing on cell proliferation. The CCK-8 assay (Fig. 3C) and colony formation assay (Fig. 3D) showed an impaired cell proliferation ability in both the LINC01605-KD Patu-8988 and SW-1990 cells compared with the control cells. We also performed Transwell assays (Fig. 3E) and wound healing assays (Fig. 3F; Supplementary Fig. 2A) on the transfected PDAC cells and the corresponding control cells, which demonstrated that LINC01605 knockdown impaired the migration ability of PDAC cells in vitro. In a complementary approach, we chose MiaPaca-2 and PANC1 cells to investigate whether overexpressing LINC01605 could enhance these cellular abilities. The two types of PDAC cells were transfected and verified to overexpress LINC01605 by qRT‒PCR (Supplementary Fig. 1A). As expected, the cellular proliferation and migration abilities of LINC01605-OE PDAC cells were relatively enhanced (Supplementary Fig. 1B–F; Supplementary Fig. 2B). Collectively, these results suggested that LINC01605 has a promoting effect on PDAC cell proliferation and migration in vitro.

### LINC01605 contributes to PDAC cell proliferation and metastatic invasion by activating the mTOR signaling pathway

To further explore how LINC01605 regulates the malignant biological behaviors of PDAC cells, we initially performed RNA-seq on Patu-8988 cells with verified LINC01605-knockdown and the respective controls (with three replicates per condition) (Fig. 3A, B). The top Kyoto Encyclopedia of Genes and Genomes (KEGG) pathways were screened out, and the mTOR signaling pathway was significantly enriched (Fig. 3C); this result was also supported by the gene set enrichment analysis (GSEA) results (Supplementary Fig. 3A, B). To verify the regulatory effect of LINC01605 on the mTOR signaling pathway, we examined the expression levels of phospho-mTOR (Ser2481) (henceforth referred to as p-mTOR) and phospho-p70 S6 kinase (Thr421/Ser424) (henceforth referred to as p-S6K) related to their corresponding total protein (total-mTOR and total-p70 S6K) levels by western blotting. As anticipated, LINC01605 knockdown led to a decrease in p-mTOR and p-S6K levels in Patu-8988 and SW-1990 cells (Fig. 3D), while LINC01605 overexpression increased the phosphorylation levels of the two key factors in MiaPaca-2 and PANC1 cells (Fig. 3E). In complementary, we treated LINC01605-overexpressing and control PDAC cells with the mTOR inhibitor rapamycin to evaluate the possible influence on cellular function. Conceivably, the previously observed enhancements in cellular proliferation (Fig. 3F, G) and migration ability (Fig. 3H, I; Supplementary Fig. 2C) were almost completely abolished through mTOR signaling blockade by rapamycin, and the phosphorylation of mTOR and S6K was inhibited (Fig. 3J). These results provide evidence that LINC01605 facilitates the malignant biological behaviors of PDAC cells by activating the mTOR signaling pathway.

### LINC01605 regulates cellular cholesterol biosynthesis in PDAC cells

**Fig. 4:**
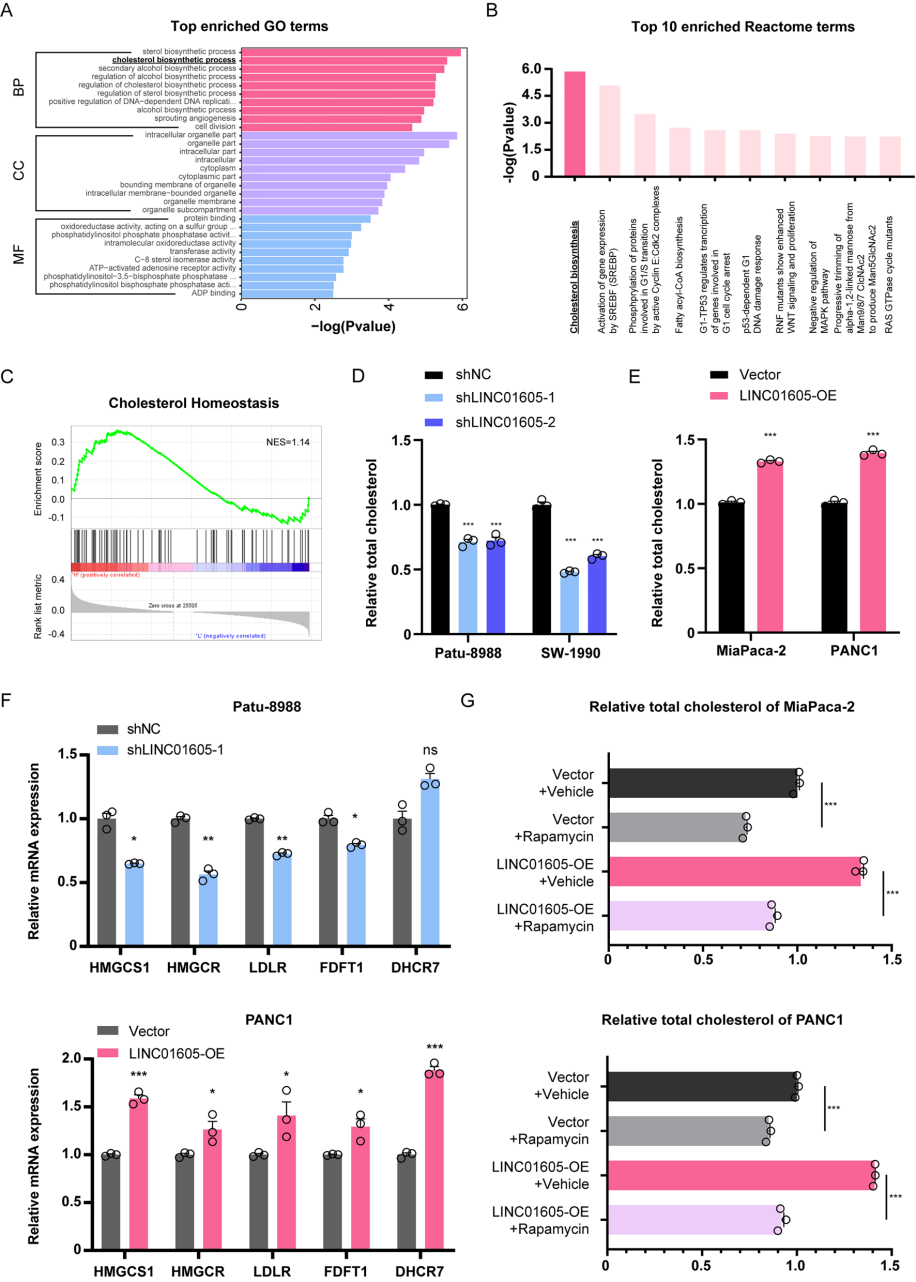
LINC01605 regulates the metabolic process of cholesterol biosynthesis of the PDAC cells. **A** The significantly enriched GO histograms of the RNA-seq samples based on P value. **B** The top 10 significantly enriched Reactome pathways ranked by −log (P value). **C** The result relative with cholesterol metabolism of gene set enrichment analysis (GSEA) by hallmark gene sets comparing the LINC01605-high and LINC01605-low expression groups of the TCGA PAAD datasets. NES (normalized enrichment score) = 1.14. **D**, **E** The relative total cellular cholesterol level of LINC01605-NC/LINC01605-KD (**D**) and Vector/LINC01605-OE (**E**) PDAC cells detected by the specific kit and measured by absorbance at 560 nm. Knockdown of LINC01605 partly restrained the cholesterol biosynthetic metabolism of the cells (**D**), while overexpression of LINC01605 enhances the effect (**E**). **F** The relative mRNA expression of five key factors in the process of cholesterol biosynthesis of the LINC01605-NC/KD Patu-8988 and Vector/LINC01605-OE PANC1 detected by qRT-PCR assay. **G** The relative total cellular cholesterol level of the two Rapamycin treated (50 nM, 48 h) LINC01605-OE and corresponding Vector cell lines showed the blocking effect by mTOR signaling inhibition on cholesterol biosynthesis

We also noticed that there was a significant enrichment in the process of cholesterol biosynthesis in the Gene Ontology (GO) analysis (Fig. 4A) and Reactome metabolic pathway analysis based on our RNA-seq data (Fig. 4B); cholesterol homeostasis was also enriched in the GSEA (Fig. 4C). As mentioned previously, the mTOR signaling pathway plays a well-established role in metabolism regulation or even reprogramming of cancer cells, while the process of PDAC tumor growth and liver metastasis consumes and demands a considerable amount of nutrients, one of which is cholesterol [23–27]. Therefore, we postulated that LINC01605 might facilitate PDAC progression via its regulatory influence on cholesterol metabolism, a downstream consequence of mTOR signaling activation. To preliminarily validate our hypothesis, we quantified total cholesterol levels in LINC01605-knockdown and LINC01605-overexpressing PDAC cells. Aligning with expectations, we observed a relative decrease in the total cellular cholesterol level of the LINC01605-knockdown PDAC cells (Fig. 4D) and an increase in cholesterol biosynthesis upon LINC01605 overexpression (Fig. 4E). Next, we examined the relative mRNA levels of five key factors within the cholesterol biosynthesis process, namely, HMGCS1, HMGCR, LDLR, FDFT1 and DHCR7 [26, 28–31], in our PDAC cells. We noticed that most of these factors were positively correlated with LINC01605, which partly elucidated the regulatory effect of LINC01605 in cholesterol biosynthesis (Fig. 4F). To further investigate whether the activation of mTOR signaling plays a role in cholesterol metabolic regulation, we again treated LINC01605-overexpressing MiaPaca-2 and PANC1 cells with rapamycin and observed an inhibitory effect reflected by the decreased total cellular cholesterol level (Fig. 4G). To ascertain the involvement of LINC01605 and mTOR signaling in cholesterol metabolic control, we carried out multi-label immunofluorescence staining on the differently-treated Patu-8988 cell climbing sheets. As shown in Supplementary Fig. 4B, a synchronous change in LINC01605 expression level and intracellular total cholesterol level could be observed. In regard of mTOR signaling pathway, the LINC01605 RNA, p-mTOR and SREBP2 protein marked in the Patu-8988 cells demonstrated a possible synergy, which provide further evidence for the potential regulation of LINC01605 on cellular cholesterol (Supplementary Fig. 4C).

In summary, these data indicate that LINC01605 regulates metabolic cholesterol biosynthesis through the mTOR signaling pathway.

### LIN28B mediates the crosstalk between LINC01605 and the mTOR signaling pathway

**Fig. 5:**
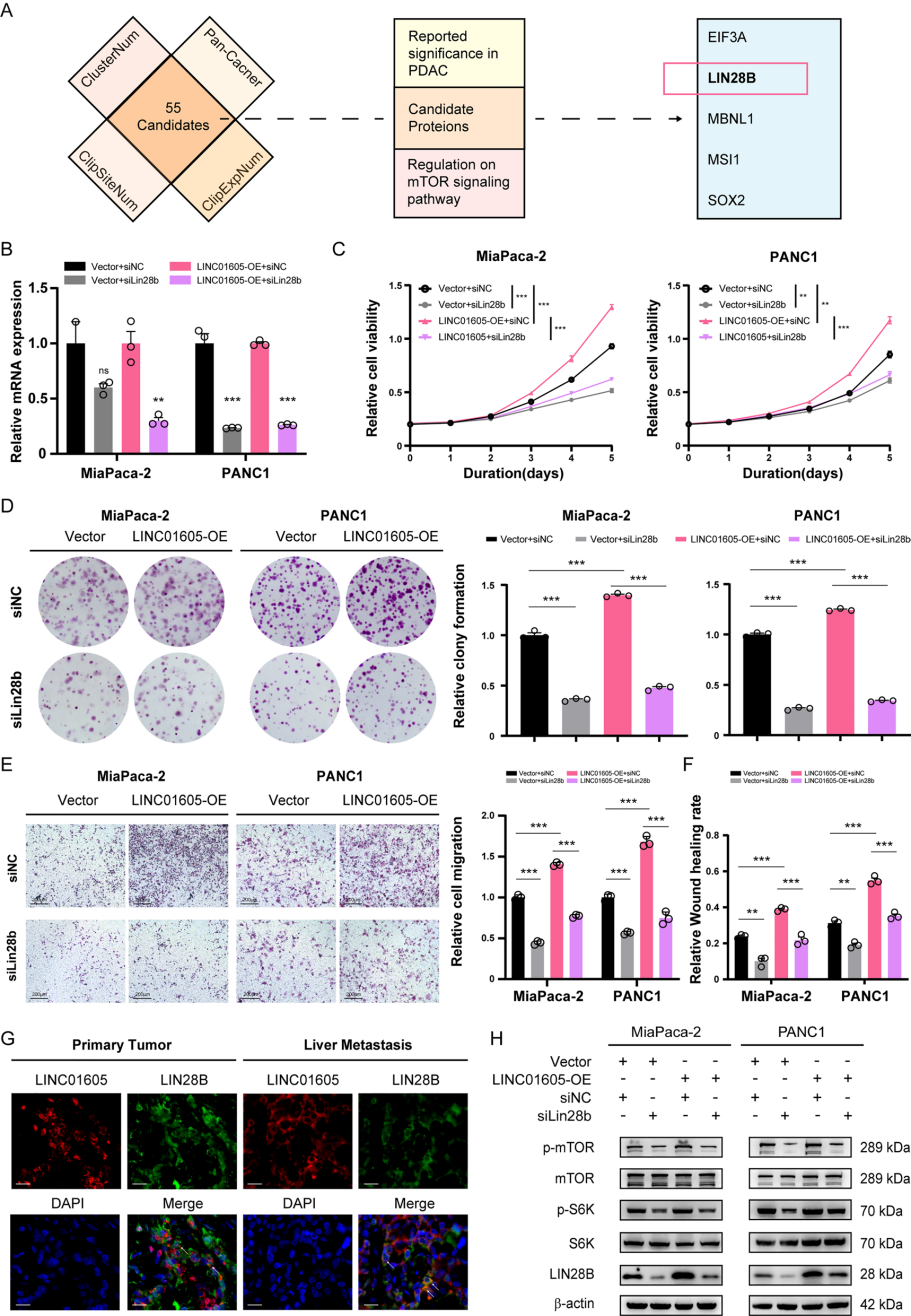
LINC01605 interacts with LIN28B in mTOR signaling transduction. **A** Schematic diagram of the screening process of the potential interacting proteins of LINC01605. ClusterNum: number of stacked regions among CLIP-seq datasets; ClipSiteNum: number of supported binding sites from CLIP-seq experiments; ClipExpNum: number of supported CLIP-seq experiments; Pan-Cancer, number of cancers (P value ≤ 0.05). **B** The related mRNA expression level of Lin28b of the siLIN28b-transfected Vector or LINC01605-OE MiaPaca-2 and PANC1 detected by qRT-PCR assay. **C** The CCK-8 assay demonstrated that the previously enhanced cellular proliferation of LINC01605-OE MiaPaca-2 and PANC1 was blocked by the effect of Lin28b knockdown. **D** The colony-formation assay demonstrated that the cellular proliferation function of LINC01605-OE MiaPaca-2 and PANC1 was partly blocked by Lin28b knockdown. **E** The Transwell assay showed the inhibited ability of cell migration of the LINC01605-OE MiaPaca-2 and PANC1 by Lin28b knockdown. Scale bar, 200 μm. **F** The 24-h relative rate of wound healing of the siNC- or siLin28b-transfected PDAC cells showed the blocking effect on migration of Lin28b knockdown. **G** Representative co-localization images of LINC01605 and LIN28B in PDAC cells within human PDAC primary tumor and liver metastasis tissues. Scale bar, 20 μm. **H** The Western-blotting assay showed the expression level of LIN28B, phosphorylated-mTOR, total mTOR, phosphorylated-S6K, total-S6K and β-actin of siNC- or siLIN28b-transfected Miapaca-2 and PANC1 groups

As a lncRNA, LINC01605 possesses inherent potential to regulate PDAC cells. However, we further asked how LINC01605 participates in the regulation of the mTOR signaling pathway. In other words, there may be a specific protein that acts as a mediator between LINC01605 and the mTOR signaling pathway. Therefore, we first searched for candidate proteins with the potential to interact with LINC01605 by ENCORI/starBase (https://rnasysu.com/encori/index.php). As illustrated in Fig. 5A, 55 RNA-binding proteins (RBPs) were filtered out based on multiple CLIP-seq data. Subsequently, we noticed among these candidates that Lin-28 homolog B (LIN28B), which is mainly detected both in the cytoplasm [32] and nucleus [33], is widely reported as a potent regulator in PDAC as well as in the mTOR signaling pathway [34–41]. These preliminary data fortified our hypothesis of a direct interaction between LIN28B and LINC01605. Therefore, we focused on LIN28B for further investigation.

To verify the interaction between LINC01605 and LIN28B, we first silenced Lin28b with siRNA in the previously constructed LINC01605-overexpressing and Vector MiaPaca-2 and PANC1 cells for subsequent experiments (Fig. 5B). As expected, the CCK-8 (Fig. 5C) and colony formation assays (Fig. 5D) showed that proliferation was greatly suppressed by silencing Lin28b, while the Transwell (Fig. 5E) and wound healing assays (Fig. 5F; Supplementary Fig. 2D) demonstrated an almost parallel inhibitory effect on cellular migration, which was previously enhanced in these cells; this effect was similar to that of rapamycin treatment. Next, we assessed whether LINC01605 was colocalized with LIN28B by immunofluorescence staining of tissue slides from PDAC patients. The results displayed the overlapping signals of LINC01605 and LIN28B as for possible colocalization of LINC01605 and LIN28B within the primary PDAC cells and the liver metastatic cells, supporting the interaction between LINC01605 and LIN28B (Fig. 5G). Importantly, the western blotting results showed that mTOR and S6K phosphorylation levels were decreased as LIN28B levels decreased in response to LINC01605 overexpression (Fig. 5H). In summary, these results suggest that LIN28B is likely involved in mediating the process by which LINC01605 triggers the mTOR signaling pathway.

### LINC01605 facilitates PDAC tumor growth and liver metastasis in vivo

**Fig. 6:**
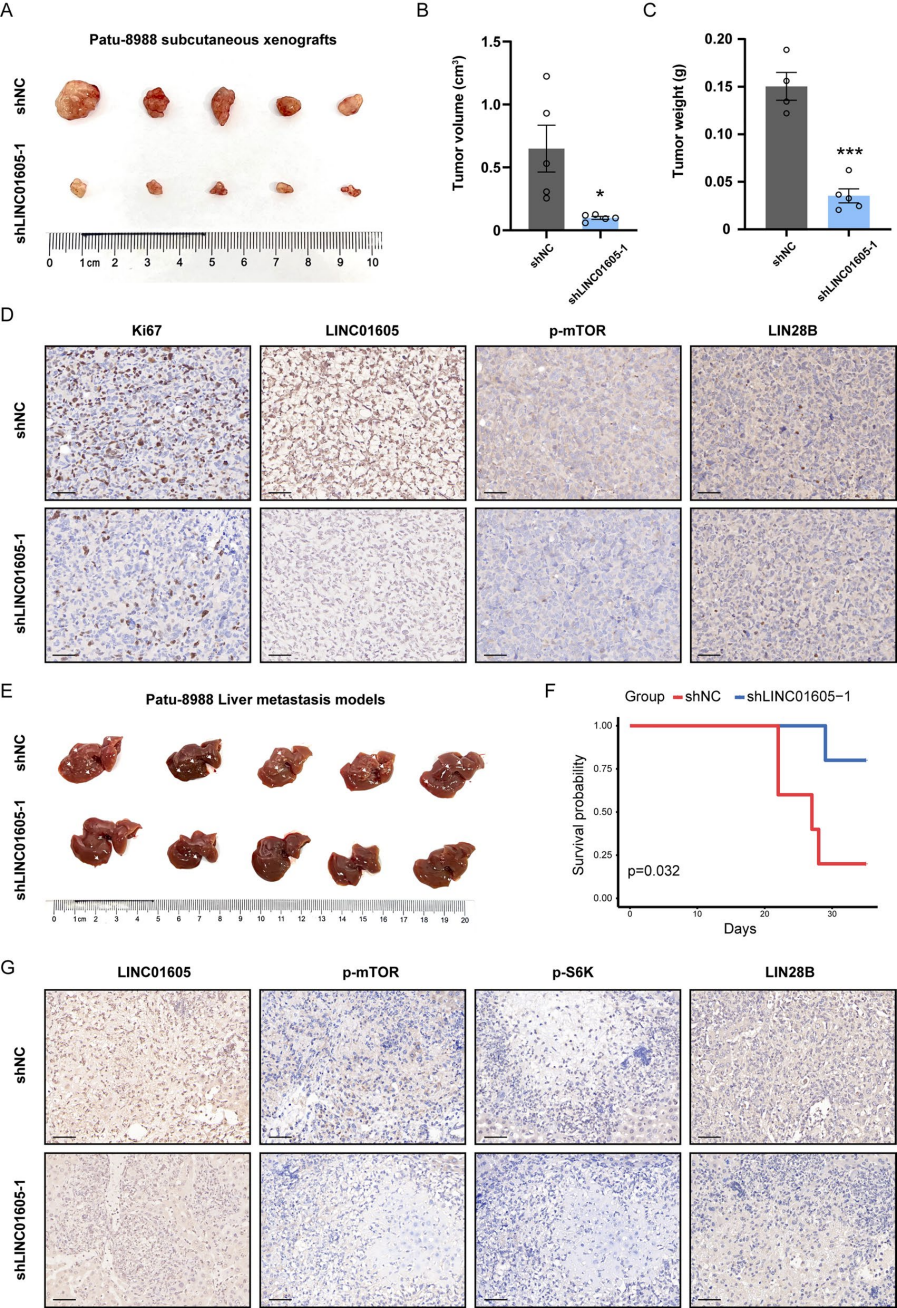
Suppressed LINC01605 expression inhibited PDAC tumor growth and liver metastasis in vivo. **A**–**C** The gross specimen image (**A**), their corresponding tumor volume (**B**) and weight (**C**) of the subcutaneous xenografts harvested from the mouse models injected with LINC01605-NC and LINC01605-KD Patu-8988 cells. **D** Representative IHC staining images of the subcutaneous xenografts for Ki67, LINC01605, p-mTOR and LIN28B. Scale bar, 50 μm. **E** The gross specimen of the livers showing the metastatic stage of LINC01605-NC and LINC01605-KD Patu-8988 cells. **F** The Kaplan–Meier analysis on survival of the parallel paired group of mouse liver metastasis models injected with LINC01605-NC and LINC01605-KD Patu-8988. p = 0.032. **G** Representative IHC staining images of the liver metastasis tissues for LINC01605, p-mTOR, p-S6K and LIN28B. Scale bar, 50 μm

**Fig. 7:**
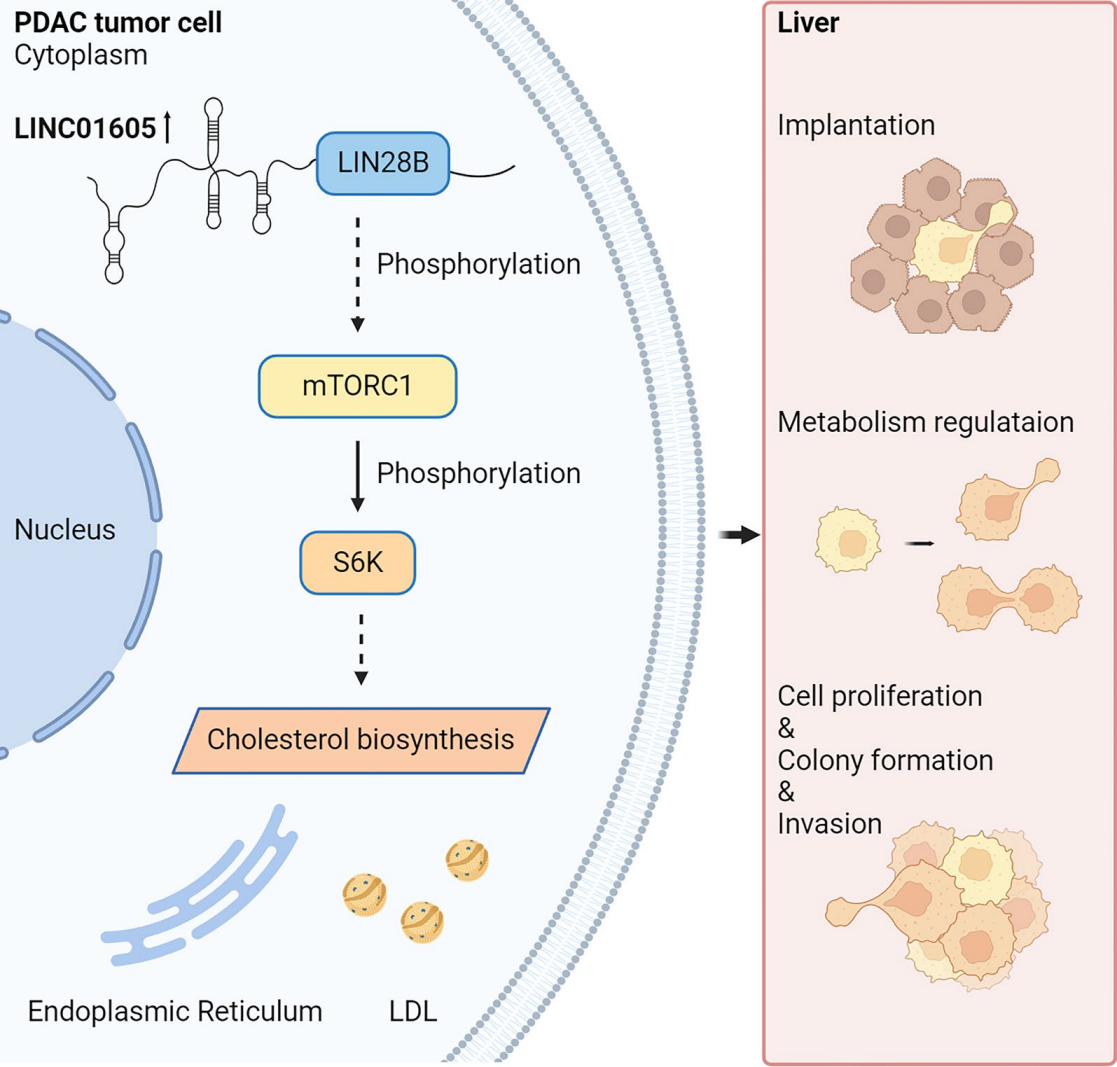
The mechanism diagram illustrating how LINC01605 promotes PDAC cell proliferation and metastatic ability. In PDAC, the abnormally up-regulated lncRNA LINC01605 interacts with LIN28B to activate the mTOR signaling pathway via mTOR phosphorylation, which then phosphorylates the p70 S6 Kinase to further positively regulate the metabolic process of cholesterol biosynthesis of the PDAC cells. In result, the PDAC cells with high LINC01605 expression gain more prominent ability on cellular proliferation, migration and metastatic invasion, which finally manifests as the process of tumor growth and liver metastasis

Confirming the regulatory effect of LINC01605 on PDAC tumor growth and liver metastasis in a living organism is of paramount significance. Therefore, we introduced two animal models, a subcutaneous xenograft model and an intrasplenic injection liver metastasis model, in our study to investigate the possible effects of LINC01605 on cell proliferation, invasion and metastasis. As shown in Fig. 6A, the visual diminution in tumor sizes of the subcutaneous xenograft models intuitively reflected the restricted cellular proliferation of the LINC01605-knockdown Patu-8988 cells. Quantitatively, the tumor volume (Fig. 6B) and weight (Fig. 6C) were both significantly lower in the LINC01605-KD group than in the control group. Additionally, we carried out IHC staining and observed weaker reactivity of the proliferation marker Ki-67, as well as LINC01605, p-mTOR and LIN28B (Fig. 6D). The results of the paired LINC01605-OE group also supported the same argument (Supplementary Fig. 5A–D). Furthermore, we successfully verified the regulatory effect of LINC01605 on metastatic invasion of the liver in liver metastasis mouse models. Figure 6E illustrates the variable extent of liver invasion in the two groups treated with LINC01605-knockdown and control Patu-8988 cells. The survival analysis of these mouse groups shows survival over time as the PDAC progressed/metastasized (Fig. 6F). In the IHC assays, indexes including LINC01605, p-mTOR, p-S6K and LIN28B were stained to estimate the metastatic ability. These factors were also downregulated in vitro (Fig. 6G). In addition, the corresponding results from the vector/LINC01605-OE group provided additional evidence that LINC01605 facilitates the liver metastatic invasion ability of PDAC cells (Supplementary Fig. 5E–G). Taken together, these results reveal that LINC01605 facilitates tumor growth and liver metastasis of PDAC in vivo, which results in tumor growth and liver metastasis (Fig. 7).

## Discussion

For years, PDAC has long been notorious as one of the most lethal malignancies and is characterized by a remarkably high incidence of local progression and liver metastasis [42, 43]. In particular, the aggressive process of PDAC liver metastasis is recognized as a major contributor to the limitations of treatments for the disease and thus leads to an unfavorable prognosis of PDAC patients [44]. For instance, the early dissemination of tumor cells, high frequency of gene mutations, the immunosuppressive microenvironment of the organ, and molecular heterogeneity and others altogether lead to the progression of PDAC in the form of liver metastasis [45–51]. LncRNAs are non-negligible factors fueling the proliferation or metastasis of tumor cells [52, 53]. Here, we provide insights into the mechanisms by which a specific lncRNA promotes PDAC tumor progression. The lncRNA LINC01605 activates the mTOR signaling pathway by interacting with LIN28B to regulate cholesterol metabolism, which facilitates PDAC cell proliferation and migration.

Given the importance of lncRNA in PDAC progression, we directed our attention to identifying a previously uncharacterized yet potentially influential lncRNA implicated in this disease process. As described above, LINC01605 emerged as a carefully selected candidate. As a promising lncRNA, LINC01605 has been studied in breast cancer [10], colorectal cancer [8], bladder cancer [9], etc.; in these tumors, LINC01605 can promote cell proliferation and migration. However, LINC01605 has not been studied in the field of pancreatic cancer, so we performed integrated analysis of clinical samples and RNA sequencing data and performed cellular function experiments and mouse model experiments to assess the role of LINC01605 in PDAC progression. Notably, in addition to the subcutaneous xenograft mouse model investigating cell proliferation in vivo, the PDAC cell intrasplenic injection mouse model we introduced to study alterations in liver and metastatic tissues has great advantages and authority [21, 54]. Our utilization of human PDAC cell-engrafted nude mice further augmented the clinical relevance and robustness of our findings, solidifying LINC01605 as a crucial determinant in the context of human PDAC progression.

To date, LINC01605 has been reported to participate in miR-3960/SOX11 [8] and METTL3/SPTBN2 regulation [12] and to play a role in the EMT signaling pathway with MMP9 [9] and the NF-κB pathway [13] or metabolic processes such as aerobic glycolysis [10]. Despite these findings, whether LINC01605 regulates tumor cells via the mTOR signaling pathway remains unclear. Numerous previous studies have reported the mTOR signaling pathway as a key factor in tumor cell metabolism regulation. Notably, the tumor microenvironment of primary PDAC or metastatic niches is characterized by hypoxia, low pH, and nutritional deprivation, and the processes of tumor growth and liver metastasis themselves are also closely dependent on metabolic regulation [23–25, 55, 56]. Our team's prior work has emphasized the heightened activation of the mTOR pathway in gastrointestinal tumors, where it governs metabolism, fostering cell survival and proliferation [20, 57]. Motivated by these factors, we explored whether and how LINC01605 regulates mTOR signaling to elucidate the underlying mechanism of the regulatory effect of LINC01605 on PDAC cell proliferation and migration. Given the enrichment of cholesterol metabolism pathways and the well-established role of mTOR in cholesterol homeostasis [26, 27, 58], we further scrutinized the involvement of cholesterol metabolism in LINC01605's regulatory effects. As expected, the phosphorylation/activation of mTOR signaling pathway components and the cell cholesterol levels were dynamically regulated as LINC01605 expression changed. These results confirmed our hypothesis that LINC01605 participates in the mTOR signaling pathway and thus regulates cholesterol metabolism, which may illustrate its mechanism for regulating the malignant behaviors of PDAC tumor cells. However, the regulatory effect of LINC01605 on cholesterol metabolism regulation requires further study, which should include cholesterol supplementation experiments.

We further filtered out LIN28B to pursue a possible link between LINC01605 and the mTOR signaling pathway. Notably, nucleic acid binding and RNA binding were included in the GO terms related to LIN28B, which provides a reasonable explanation for the fact that LIN28B mainly interacts with microRNAs such as let-7 family members [33, 37, 40, 41]. Recognizing LIN28B's propensity for miRNA interaction, we posited its potential to collaborate not only with miRNAs but also with lncRNAs, thereby amplifying regulatory signals from its interaction partners. Conventionally, lncRNAs are frequently reported to interact with miRNAs, while interactions between a lncRNA and a certain protein are rather rare. However, as a bridge with the innate ability to bind RNAs and a key regulator in the mTOR signaling pathway, LIN28B appears to be one of the most likely interacting proteins for LINC01605 within the mTOR signaling pathway, as there have been reports that LINC01094 sponges miR-577 to regulate LIN28B and the PI3K/AKT pathway [38]. Our study provides evidence to some extent of the interaction between LINC01605 and LIN28B and its role in regulating cell proliferation, invasion and metastasis through the mTOR signaling pathway based on bioinformatics predictions, immunofluorescence colocalization analysis, rescue experiments and corresponding protein-level verification. Consequently, LIN28B has been characterized as a lncRNA LINC01605-interacting protein, illuminating a multifaceted mechanism underlying LINC01605-driven PDAC tumor progression and liver metastasis. However, additional studies are warranted to conclusively validate the direct interaction between LINC01605 and LIN28B, fortifying our understanding of this intricate regulatory axis in PDAC biology.

In conclusion, we identified LINC01605 as a key player in regulating the proliferation, invasion and metastasis of PDAC cells; it interacts with LIN28B and activates the mTOR signaling pathway to regulate cholesterol metabolism. By this mechanism, PDAC cells with LINC01605 upregulation acquire advantages that facilitate their progression based on the effects on cholesterol metabolism regulation. Moreover, our study further supports the usage of mTOR inhibitors in PDAC and suggests the clinical value of LINC01605 as a prognostic index as well as an accessory therapeutic target for PDAC intervention.

## Conclusions

Our results show that lncRNA LINC01605, which is markedly upregulated in PDAC primary tumor and liver metastasis tissues, promotes PDAC progression in by facilitating tumor cell proliferation and metastatic invasion through cholesterol metabolism regulation by activating the mTOR signaling pathway in a LIN28B-interacting manner. Collectively, our study provides new insight into the role of LINC01605 in PDAC tumor growth and liver metastasis, which suggests its clinical value against the deadly disease.

### Supplementary Information

Supplementary Material 1.

## Data Availability

The datasets used and/or analyzed during the current study are available from the corresponding author on reasonable request.

## Abbreviations

PDAC: Pancreatic ductal adenocarcinoma
lncRNA: Long noncoding RNA
LINC01605: Long intergenic non-protein coding RNA 1605
mTOR: Mammalian/mechanist target of rapamycin
SREBP2, also known as SREBF2: Sterol Regulatory Element Binding Transcription Factor 2
LIN28B: Lin-28 homolog B
TCGA: The Cancer Genome Atlas
DFS: Disease-free survival
RNA-seq: RNA sequencing
S6K: P70 S6 kinase
TMA: Tumor Micro Array
H&E: Hematoxylin and eosin
IHC: Immunohistochemistry
IF: Immunofluorescence
PBS: Phosphate buffer solution
ATCC: American Type Culture Collection
FBS: Fetal bovine serum
P/S: Streptomycin/penicillin
qRT-PCR: Quantitative real-time polymerase chain reaction
WB: Western blotting
NC: Negative control
shRNA: Short hairpin RNA
siRNA: Small interfering RNA
CCK-8: Cell Counting Kit-8
SEM: Standard error of the mean
DEG: Differentially expressed gene
GSEA: Gene set enrichment analysis
GO: Gene Ontology
RBP: RNA-binding protein
