# Supplementary material for: The lncRNA LINC01605 promotes the progression of pancreatic ductal adenocarcinoma by activating the mTOR signaling pathway
Source: Cancer Cell Int. 2024 Jul 24;24:262. doi: 10.1186/s12935-024-03440-z (PMC11271012; doi:10.1186/s12935-024-03440-z)
Supplement: Supplementary file 1 — Supplementary Material 1. [file 12935_2024_3440_MOESM1_ESM.docx]

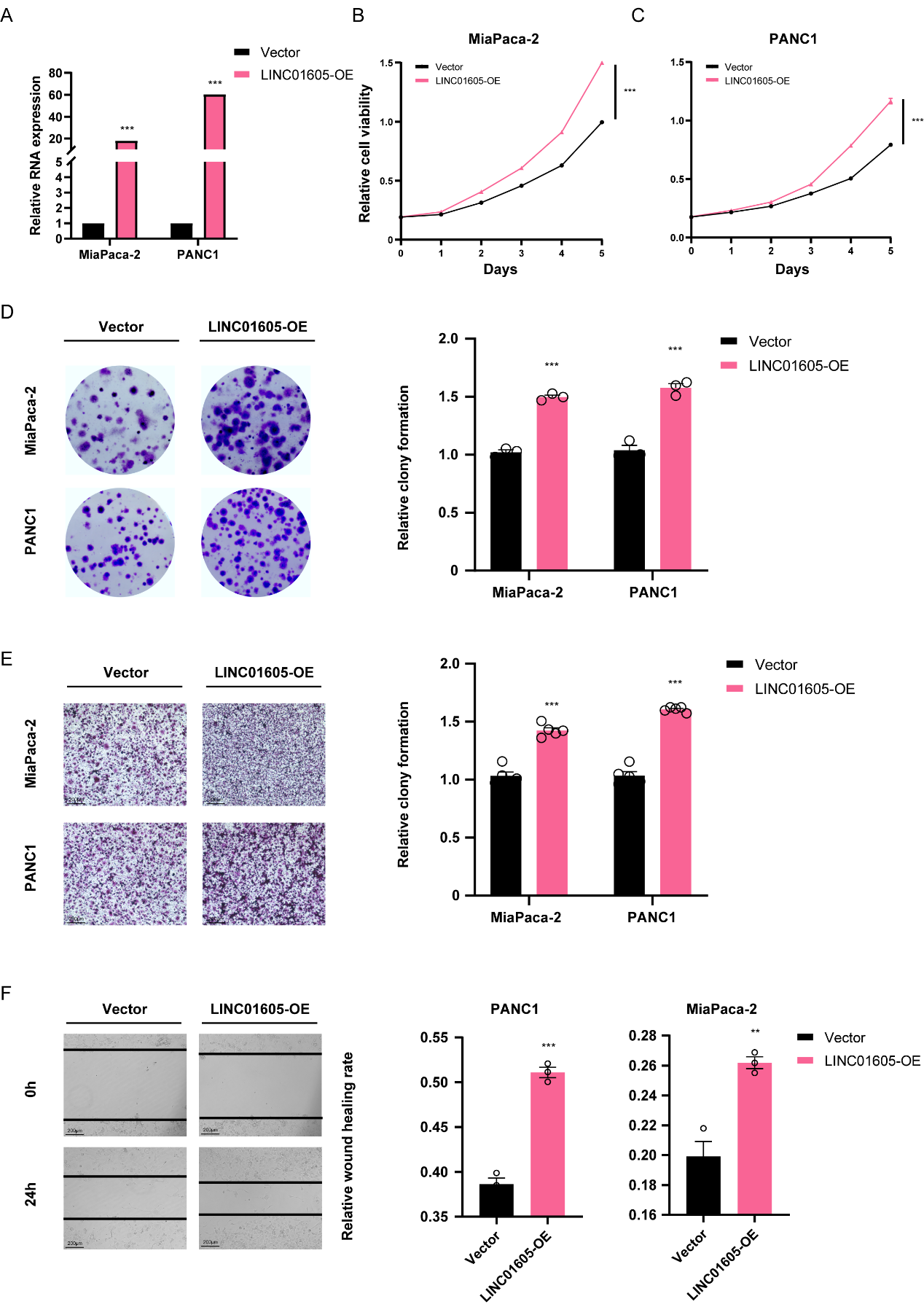


Supplementary Figure 1. Overexpression of LINC01605 enhances the cellular function of PDAC cell proliferation and migration *in vitro*.

A. The relative mRNA expression of LINC01605 in LINC01605-Overexpression (LINC01605-OE) and corresponding vector (Vector) PDAC cells MiaPaca-2 and PANC1 by lentivirus transfection.

B-C. The CCK-8 assay demonstrated that the proliferation ability of MiaPaca-2 and PANC1 cells was enhanced by the effect of LIC01605 overexpression.

D. The colony-formation assay demonstrated that the LINC01605-OE MiaPaca-2 and PANC1 had more remarkable ability of colony formation as a form of proliferation.

E. The Transwell assay reflected the enhanced cellular function of migration of the LINC01605-OE MiaPaca-2 and PANC1.

F. The 24-hour wound healing assay of the indicated PDAC cells demonstrated the relatively enhanced ability of migration of LINC01605-OE cells. Scale bar, 200μm. (PANC1 shown as representative images)


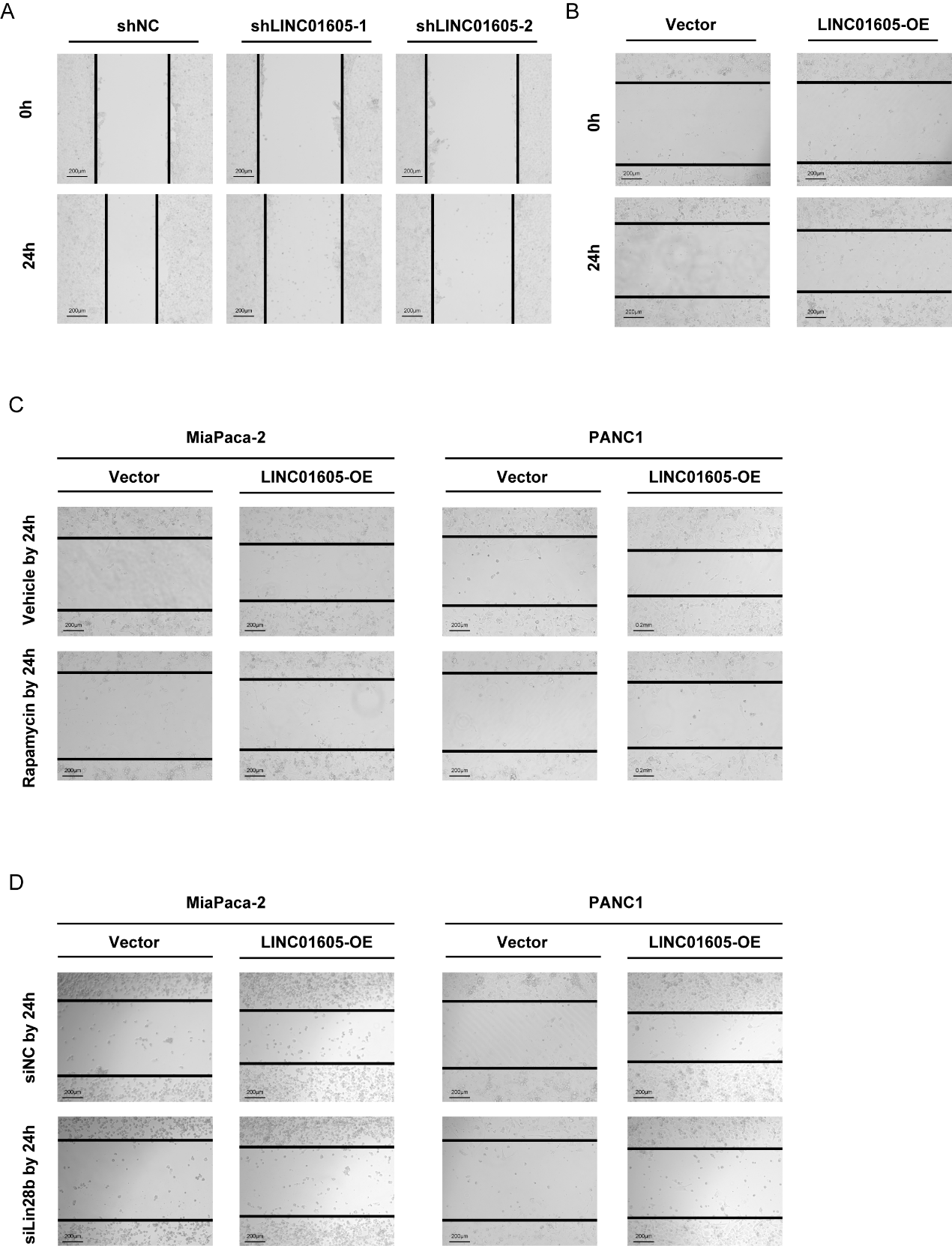


Supplementary Figure 2. Representative images of wound healing assay snapped on the timepoint of 0h and 24h.

A. Wound healing situation of shNC, shLINC01605-1 and shLINC01605-2 SW-1990.

B. Wound healing situation of Vector and LINC01605-OE MiaPaca-2.

C. Representative wound healing images of Vector and LINC01605-OE MiaPaca-2/PANC1 treated with Rapamycin or DMSO as the vehicle.

D. Representative wound healing images of Vector and LINC01605-OE MiaPaca-2/PANC1 transfected with siNC or siLin28b.

(Scale bar in Supplement Figure.2., 200μm.)


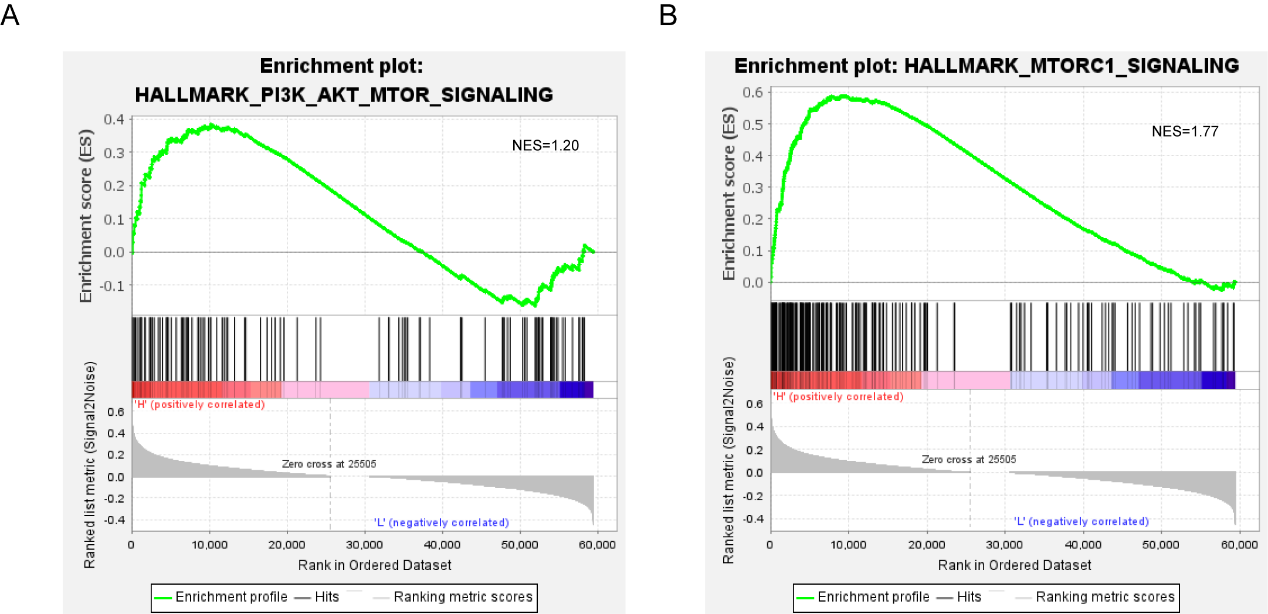


Supplementary Figure 3. GSEA (Gene Set Enrichment Analysis) relative with mTOR signaling pathway by hallmark gene sets.

A. GSEA results of PI3K-AKT-mTOR signaling. NES (normalized enrichment score)=1.20.

B. GSEA results of mTOC1 signaling. NES=1.77.


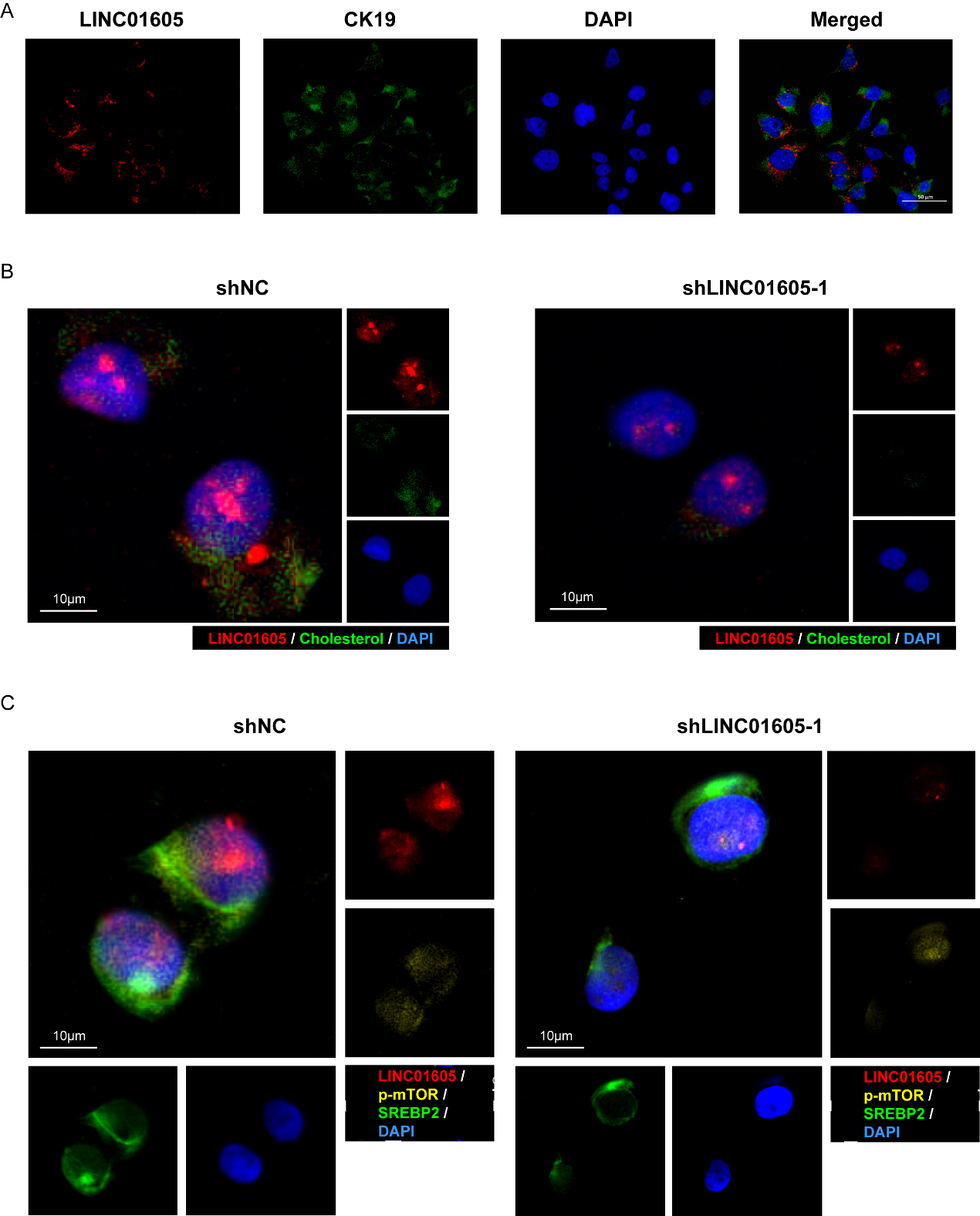


Supplementary Figure 4. IF results in differently-treated Patu-8988 cells reveal the pattern and possible mechanism of LINC01605.

A. Representative Immunofluorescence images of LINC01605, CK19, DAPI and the merged image in PDAC cell line Patu-8988. Scale bar, 50μm.

B. Representative IF staining images from shNC- and shLINC01605-1-transfected Patu-8988 cells on LINC01605 and cholesterol. Scale bar, 10μm.

C. Representative IF staining images from shNC- and shLINC01605-1-transfected Patu-8988 cells on LINC01605, p-mTOR and SREBP2. Scale bar, 10μm.


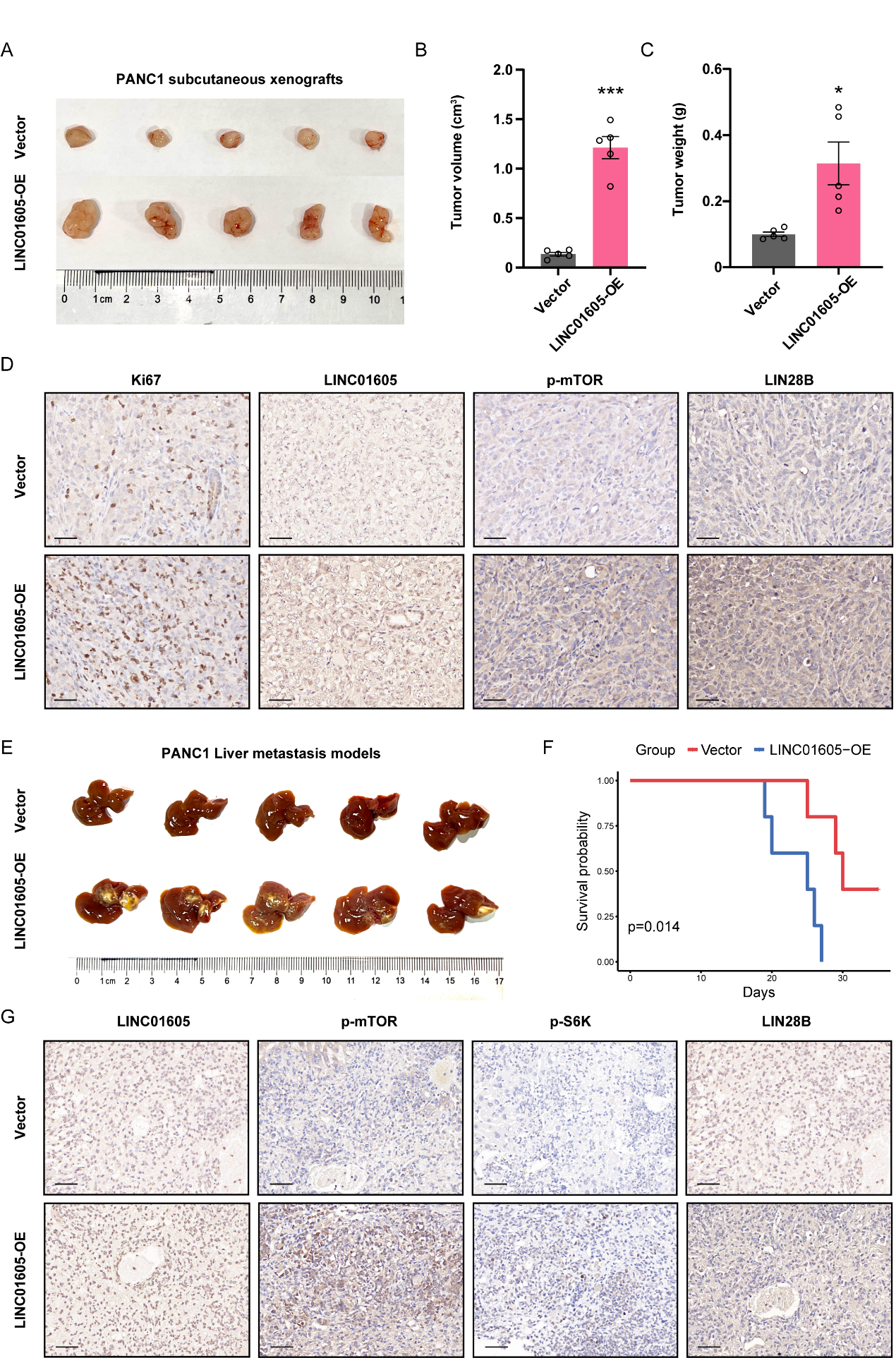


Supplementary Figure.5. Overexpressed LINC01605 promoted PDAC tumor growth and liver metastasis *in vivo*.

A-C. The gross specimen image (A), their corresponding tumor volume (B) and weight (C) of the subcutaneous xenografts harvested from the mouse models injected with Vector and LINC01605-OE PANC1 cells.

D. Representative IHC staining images of the subcutaneous xenografts for Ki67, LINC01605, p-mTOR and LIN28B. Scale bar, 50μm.

E. The gross specimen of the livers showing the early stage of liver metastasis of Vector and LINC01605-OE PANC1 cells.

F. The Kaplan-Meier analysis on survival of the parallel paired group of mouse liver metastasis models injected with Vector and LINC01605-OE PANC1. p=0.014.

G. Representative IHC staining images of the liver metastasis tissues for LINC01605, p-mTOR, p-S6K and LIN28B. Scale bar, 50μm.

Supplementary Table 1. The PDAC TMA with clinical indices.

| **P Num.** | **Sex** | **A.** | **P.** | **V.** | **L.** | **Diff.** | **TNM** | **LM.** | **DM.** | **OS.** | **SS.** | **Score** | **Group** |
| --- | --- | --- | --- | --- | --- | --- | --- | --- | --- | --- | --- | --- | --- |

| 17-25152 | 1 | 69 | 2 | 0 | 2.5 | 0 | 1 | 1 | 1 | 758 | 1 | 6 | 1 |
| --- | --- | --- | --- | --- | --- | --- | --- | --- | --- | --- | --- | --- | --- |
| 17-14678 | 1 | 74 | 2 | 1 | 3.5 | 1 | 1 | 1 | 1 | 543 | 1 | 6 | 1 |
| 18-11726 | 0 | 61 | 2 | 1 | 4.5 | 0 | 1 | 1 | 1 | 467 | 1 | 9 | 1 |

| 17-15420 | 1 | 47 | 1 | 0 | 0.5 | 0 | 1 | 1 | 1 | 333 | 1 | 9 | 1 |
| --- | --- | --- | --- | --- | --- | --- | --- | --- | --- | --- | --- | --- | --- |
| 18-21555 | 1 | 63 | 1 | 1 | 3 | 1 | 1 | 1 | 1 | 253 | 1 | 9 | 1 |
| 17-20298 | 0 | 83 | 1 | 0 | 2.5 | 1 | 1 | 1 | 1 | 154 | 1 | 9 | 1 |
| 17-19077 | 1 | 76 | 1 | 1 | 3 | 1 | 1 | 1 | 1 | 126 | 1 | 6 | 1 |
| 17-19104 | 0 | 54 | 1 | 0 | 2.5 | 1 | 1 | 1 | 0 | 495 | 1 | 6 | 1 |
| 17-24580 | 0 | 65 | 1 | 0 | 2 | 1 | 1 | 1 | 0 | 458 | 1 | 9 | 1 |
| 18-36445 | 0 | 63 | 2 | 1 | 5 | 1 | 1 | 1 | 0 | 295 | 1 | 9 | 1 |
| 17-04588 | 1 | 63 | 1 | 0 | 2.5 | 1 | 1 | 1 | 0 | 281 | 1 | 6 | 1 |
| 17-06184 | 0 | 84 | 2 | 0 | 2.5 | 1 | 1 | 1 | 0 | 216 | 1 | 6 | 1 |
| 17-07813 | 0 | 70 | 1 | 1 | 4.5 | 1 | 1 | 1 | 0 | 185 | 1 | 6 | 1 |
| 18-36448 | 1 | 73 | 2 | 1 | 5 | 1 | 1 | 1 | 0 | 185 | 1 | 6 | 1 |
| 18-12885 | 1 | 78 | 1 | 1 | 4.5 | 1 | 1 | 1 | 0 | 125 | 1 | 6 | 1 |
| 17-09463 | 1 | 64 | 1 | 1 | 3 | 1 | 1 | 1 | 0 | 248 | 1 | 6 | 1 |
| 17-03706 | 1 | 62 | 1 | 1 | 4.5 | 1 | 1 | 0 | 1 | 628 | 1 | 9 | 1 |
| 17-13980 | 0 | 51 | 1 | 1 | 4 | 1 | 1 | 0 | 1 | 549 | 1 | 6 | 1 |
| 18-19158 | 1 | 66 | 2 | 1 | 6.5 | 0 | 1 | 0 | 1 | 525 | 1 | 9 | 1 |
| 17-20413 | 1 | 71 | 1 | 0 | 2.8 | 0 | 1 | 0 | 1 | 493 | 1 | 6 | 1 |
| 18-26978 | 1 | 78 | 2 | 1 | 5 | 1 | 1 | 0 | 1 | 427 | 1 | 9 | 1 |
| 18-07770 | 1 | 70 | 2 | 1 | 7 | 1 | 1 | 0 | 1 | 336 | 1 | 6 | 1 |
| 18-18548 | 1 | 82 | 2 | 1 | 5 | 1 | 1 | 0 | 1 | 1826 | 0 | 9 | 1 |
| 18-09068 | 0 | 63 | 2 | 0 | 2.5 | 1 | 0 | 0 | 0 | 183 | 1 | 6 | 1 |
| 18-14942 | 1 | 73 | 1 | 1 | 3.5 | 1 | 1 | 1 | 0 | 563 | 1 | 9 | 1 |
| 17-02565 | 1 | 66 | 1 | 1 | 3.5 | 0 | 1 | 1 | 0 | 206 | 1 | 6 | 1 |
| 18-03178 | 1 | 66 | 1 | 0 | 2.5 | 1 | 1 | 1 | 0 | 507 | 1 | 6 | 1 |
| 17-11607 | 1 | 58 | 1 | 0 | 2.5 | 1 | 1 | 1 | 0 | 628 | 1 | 6 | 1 |
| 17-28153 | 0 | 47 | 2 | 1 | 5 | 0 | 1 | 1 | 0 | 395 | 1 | 6 | 1 |
| 18-04779 | 1 | 58 | 1 | 0 | 2.2 | 0 | 1 | 1 | 0 | 983 | 1 | 9 | 1 |
| 18-06655 | 0 | 57 | 1 | 0 | 2.5 | 0 | 1 | 1 | 0 | 678 | 1 | 6 | 1 |
| 18-14942 | 1 | 73 | 1 | 1 | 3.5 | 1 | 1 | 1 | 0 | 563 | 1 | 6 | 1 |
| 17-25335 | 1 | 80 | 2 | 1 | 4 | 1 | 1 | 1 | 0 | 525 | 1 | 9 | 1 |
| 18-27640 | 0 | 54 | 2 | 1 | 6 | 0 | 1 | 0 | 0 | 1743 | 0 | 6 | 1 |
| 18-17640 | 1 | 58 | 2 | 1 | 5 | 0 | 1 | 0 | 0 | 1829 | 0 | 6 | 1 |
| 17-04931 | 1 | 76 | 1 | 1 | 3.5 | 1 | 1 | 1 | 0 | 220 | 1 | 6 | 1 |
| 17-08785 | 1 | 65 | 1 | 0 | 2 | 1 | 1 | 1 | 0 | 301 | 1 | 9 | 1 |
| 18-00101 | 1 | 50 | 1 | 0 | 1.2 | 0 | 1 | 1 | 0 | 1997 | 0 | 1 | 0 |
| 17-21786 | 0 | 64 | 1 | 1 | 3 | 0 | 1 | 1 | 0 | 462 | 1 | 2 | 0 |
| 17-11445 | 0 | 65 | 1 | 1 | 3 | 0 | 1 | 1 | 0 | 435 | 1 | 2 | 0 |
| 18-19524 | 0 | 72 | 1 | 1 | 3.5 | 0 | 1 | 1 | 0 | 407 | 1 | 3 | 0 |

| 17-26683 | 1 | 64 | 1 | 1 | 3 | 0 | 1 | 1 | 0 | 1188 | 1 | 2 | 0 |
| --- | --- | --- | --- | --- | --- | --- | --- | --- | --- | --- | --- | --- | --- |
| 17-14800 | 1 | 69 | 1 | 1 | 3 | 0 | 1 | 1 | 0 | 146 | 1 | 1 | 0 |
| 17-29247 | 1 | 43 | 1 | 1 | 3.5 | 1 | 1 | 1 | 0 | 285 | 1 | 4 | 0 |
| 17-21370 | 0 | 59 | 1 | 1 | 3 | 1 | 1 | 1 | 0 | 336 | 1 | 3 | 0 |
| 18-11804 | 0 | 59 | 1 | 1 | 4 | 0 | 1 | 1 | 0 | 563 | 1 | 2 | 0 |
| 17-02383 | 0 | 85 | 1 | 0 | 1.5 | 1 | 1 | 0 | 0 | 245 | 1 | 4 | 0 |
| 17-28501 | 0 | 64 | 2 | 0 | 2.5 | 0 | 0 | 0 | 0 | 677 | 1 | 0 | 0 |
| 18-14019 | 1 | 58 | 2 | 1 | 3 | 0 | 1 | 1 | 0 | 1278 | 1 | 3 | 0 |
| 17-19107 | 0 | 76 | 1 | 1 | 3 | 1 | 1 | 1 | 0 | 1083 | 1 | 4 | 0 |
| 18-25509 | 0 | 51 | 2 | 0 | 2.5 | 0 | 1 | 1 | 0 | 1755 | 0 | 2 | 0 |
| 18-37414 | 0 | 50 | 1 | 1 | 5 | 0 | 1 | 1 | 0 | 1641 | 0 | 2 | 0 |
| 18-30845 | 0 | 75 | 1 | 1 | 4.5 | 0 | 1 | 1 | 0 | 1710 | 0 | 2 | 0 |
| 18-24100 | 0 | 68 | 2 | 1 | 5.5 | 0 | 1 | 1 | 0 | 1776 | 0 | 2 | 0 |
| 17-15029 | 1 | 68 | 2 | 1 | 9 | 0 | 1 | 1 | 0 | 547 | 1 | 3 | 0 |
| 17-11338 | 1 | 77 | 2 | 1 | 5.5 | 0 | 1 | 1 | 0 | 277 | 1 | 2 | 0 |
| 17-04982 | 1 | 72 | 1 | 0 | 2 | 1 | 0 | 0 | 0 | 453 | 1 | 4 | 0 |
| 18-12444 | 1 | 62 | 1 | 0 | 2.5 | 0 | 0 | 0 | 0 | 1210 | 1 | 4 | 0 |
| 17-28746 | 1 | 63 | 1 | 1 | 3 | 1 | 0 | 0 | 0 | 910 | 1 | 3 | 0 |
| 18-36191 | 0 | 64 | 1 | 0 | 2.5 | 0 | 0 | 0 | 0 | 732 | 1 | 4 | 0 |
| 17-30007 | 1 | 72 | 1 | 1 | 3 | 1 | 0 | 0 | 0 | 470 | 1 | 4 | 0 |
| 18-32745 | 0 | 58 | 2 | 0 | 2.5 | 0 | 0 | 0 | 0 | 1701 | 0 | 4 | 0 |
| 18-02820 | 0 | 65 | 2 | 1 | 4 | 1 | 0 | 0 | 0 | 1966 | 0 | 4 | 0 |
| 18-05018 | 0 | 58 | 2 | 1 | 3.5 | 0 | 0 | 0 | 0 | 935 | 1 | 2 | 0 |
| 18-03515 | 1 | 66 | 1 | 1 | 3 | 0 | 0 | 0 | 0 | 1961 | 0 | 1 | 0 |
| 17-17666 | 1 | 68 | 2 | 1 | 3 | 0 | 0 | 0 | 0 | 2165 | 0 | 2 | 0 |
| 18-7164 | 0 | 70 | 1 | 1 | 3 | 1 | 0 | 0 | 0 | 1914 | 0 | 4 | 0 |
| 17-24140 | 1 | 62 | 2 | 1 | 3 | 1 | 0 | 0 | 0 | 1456 | 1 | 3 | 0 |
| 17-03279 | 1 | 54 | 1 | 0 | 1.5 | 0 | 0 | 0 | 0 | 2337 | 0 | 2 | 0 |
| 17-03034 | 0 | 65 | 2 | 0 | 2 | 0 | 0 | 0 | 0 | 2320 | 0 | 2 | 0 |
| 17-02696 | 1 | 60 | 1 | 1 | 4 | 0 | 0 | 0 | 0 | 2320 | 0 | 1 | 0 |
| 17-07035 | 1 | 64 | 1 | 1 | 3.5 | 0 | 0 | 0 | 0 | 2275 | 0 | 3 | 0 |
| 17-11263 | 1 | 65 | 2 | 1 | 3 | 0 | 0 | 0 | 0 | 2249 | 0 | 2 | 0 |
| 17-13704 | 0 | 75 | 1 | 0 | 2 | 0 | 0 | 0 | 0 | 2213 | 0 | 1 | 0 |
| 17-17240 | 1 | 68 | 1 | 1 | 3 | 0 | 0 | 0 | 0 | 2192 | 0 | 3 | 0 |
| 17-23671 | 1 | 66 | 1 | 1 | 4 | 0 | 0 | 0 | 0 | 2185 | 1 | 3 | 0 |
| 17-17352 | 1 | 70 | 1 | 1 | 5 | 0 | 0 | 0 | 0 | 2166 | 0 | 1 | 0 |
| 18-04961 | 1 | 61 | 1 | 0 | 2.5 | 0 | 0 | 0 | 0 | 2128 | 0 | 3 | 0 |
| 18-11962 | 0 | 66 | 1 | 0 | 2.2 | 0 | 0 | 0 | 0 | 1879 | 0 | 3 | 0 |

| 18-11960 | 1 | 71 | 1 | 0 | 2 | 1 | 0 | 0 | 0 | 1875 | 0 | 2 | 0 |
| --- | --- | --- | --- | --- | --- | --- | --- | --- | --- | --- | --- | --- | --- |
| 18-22182 | 0 | 71 | 2 | 1 | 3.5 | 0 | 0 | 0 | 0 | 1813 | 0 | 1 | 0 |
| 18-26774 | 1 | 68 | 2 | 1 | 3 | 0 | 1 | 0 | 0 | 1781 | 0 | 1 | 0 |
| 18-23968 | 0 | 65 | 1 | 0 | 2.5 | 0 | 0 | 0 | 0 | 1766 | 0 | 1 | 0 |
| 18-33023 | 1 | 71 | 1 | 1 | 6 | 0 | 0 | 0 | 0 | 1683 | 0 | 1 | 0 |
| 18-34713 | 0 | 66 | 2 | 0 | 2.5 | 1 | 0 | 0 | 0 | 1676 | 0 | 2 | 0 |
| 18-38247 | 1 | 64 | 1 | 0 | 2.5 | 1 | 0 | 0 | 0 | 1645 | 0 | 2 | 0 |
| 17-14799 | 0 | 65 | 2 | 1 | 7 | 0 | 0 | 0 | 0 | 1539 | 1 | 2 | 0 |
| 17-18426 | 1 | 70 | 1 | 1 | 3 | 0 | 0 | 0 | 0 | 1529 | 1 | 3 | 0 |
| 17-18141 | 1 | 74 | 1 | 0 | 2.8 | 1 | 0 | 0 | 0 | 1428 | 1 | 1 | 0 |
| 17-31780 | 1 | 73 | 1 | 1 | 6 | 1 | 0 | 0 | 0 | 1377 | 1 | 2 | 0 |
| 17-32952 | 1 | 67 | 2 | 0 | 1 | 0 | 0 | 0 | 0 | 1370 | 1 | 1 | 0 |
| 17-12036 | 1 | 72 | 1 | 0 | 2.5 | 0 | 0 | 0 | 0 | 1253 | 1 | 0 | 0 |
| 18-13058 | 1 | 55 | 1 | 1 | 3.5 | 1 | 0 | 0 | 0 | 1199 | 1 | 3 | 0 |
| 17-14943 | 0 | 42 | 1 | 1 | 3.5 | 0 | 0 | 0 | 0 | 1136 | 1 | 1 | 0 |
| 18-07168 | 1 | 66 | 2 | 0 | 2.5 | 0 | 0 | 0 | 0 | 1078 | 1 | 0 | 0 |
| 18-11240 | 1 | 65 | 2 | 1 | 7 | 0 | 0 | 0 | 0 | 1014 | 1 | 2 | 0 |
| 17-28926 | 0 | 65 | 1 | 1 | 5 | 0 | 0 | 0 | 0 | 938 | 1 | 2 | 0 |
| 18-20140 | 1 | 68 | 1 | 0 | 2.5 | 0 | 0 | 0 | 0 | 921 | 1 | 1 | 0 |
| 18-03911 | 0 | 62 | 1 | 0 | 2.5 | 1 | 0 | 0 | 0 | 867 | 1 | 3 | 0 |
| 17-25236 | 0 | 87 | 1 | 1 | 4 | 0 | 0 | 0 | 0 | 861 | 1 | 2 | 0 |
| 18-07700 | 1 | 48 | 2 | 1 | 7 | 0 | 0 | 0 | 0 | 830 | 1 | 1 | 0 |
| 17-02985 | 1 | 62 | 1 | 1 | 3.5 | 1 | 0 | 0 | 0 | 792 | 1 | 2 | 0 |
| 18-25463 | 1 | 83 | 1 | 0 | 2 | 0 | 0 | 0 | 0 | 684 | 1 | 2 | 0 |
| 17-19960 | 1 | 71 | 1 | 0 | 2.5 | 0 | 0 | 0 | 0 | 684 | 1 | 2 | 0 |
| 17-20158 | 1 | 35 | 2 | 1 | 4 | 0 | 0 | 0 | 0 | 677 | 1 | 2 | 0 |
| 17-07697 | 1 | 76 | 1 | 1 | 3.5 | 0 | 0 | 0 | 0 | 629 | 1 | 2 | 0 |
| 18-21653 | 1 | 73 | 1 | 1 | 3.5 | 1 | 0 | 0 | 0 | 595 | 1 | 3 | 0 |
| 18-19845 | 1 | 70 | 1 | 1 | 3 | 1 | 0 | 0 | 0 | 573 | 1 | 3 | 0 |
| 17-33966 | 1 | 79 | 2 | 1 | 3.5 | 1 | 0 | 0 | 0 | 538 | 1 | 1 | 0 |
| 18-30186 | 1 | 74 | 1 | 1 | 3 | 0 | 0 | 0 | 0 | 529 | 1 | 3 | 0 |
| 17-19735 | 1 | 83 | 1 | 1 | 3.5 | 0 | 1 | 0 | 0 | 507 | 1 | 2 | 0 |
| 17-30994 | 1 | 47 | 2 | 1 | 3 | 0 | 0 | 0 | 0 | 506 | 1 | 2 | 0 |
| 18-03919 | 1 | 71 | 1 | 1 | 4 | 0 | 0 | 0 | 0 | 492 | 1 | 2 | 0 |
| 17-12371 | 0 | 71 | 1 | 0 | 2.5 | 1 | 0 | 0 | 0 | 455 | 1 | 1 | 0 |
| 18-30043 | 1 | 66 | 1 | 1 | 4 | 1 | 0 | 0 | 0 | 408 | 1 | 2 | 0 |
| 18-30844 | 1 | 81 | 1 | 0 | 2 | 0 | 0 | 0 | 0 | 404 | 1 | 1 | 0 |
| 17-33618 | 0 | 50 | 1 | 1 | 4 | 1 | 0 | 0 | 0 | 399 | 1 | 2 | 0 |

| 18-01078 | 1 | 64 | 1 | 1 | 6 | 0 | 0 | 0 | 0 | 373 | 1 | 3 | 0 |
| --- | --- | --- | --- | --- | --- | --- | --- | --- | --- | --- | --- | --- | --- |
| 17-24437 | 0 | 71 | 2 | 1 | 5 | 1 | 0 | 0 | 0 | 362 | 1 | 2 | 0 |
| 17-02647 | 1 | 80 | 2 | 1 | 8 | 0 | 0 | 0 | 0 | 353 | 1 | 4 | 0 |
| 18-31092 | 0 | 84 | 1 | 0 | 2 | 0 | 0 | 0 | 0 | 326 | 1 | 2 | 0 |
| 18-17259 | 1 | 83 | 1 | 1 | 3.5 | 1 | 0 | 0 | 0 | 303 | 1 | 1 | 0 |
| 18-34875 | 0 | 73 | 1 | 1 | 5.5 | 0 | 0 | 0 | 0 | 290 | 1 | 2 | 0 |
| 18-30622 | 1 | 85 | 1 | 1 | 4 | 0 | 0 | 0 | 0 | 238 | 1 | 1 | 0 |
| 17-10128 | 0 | 73 | 1 | 1 | 6 | 1 | 0 | 0 | 0 | 221 | 1 | 3 | 0 |
| 18-06808 | 1 | 71 | 1 | 1 | 3 | 0 | 0 | 0 | 0 | 210 | 1 | 0 | 0 |
| 18-26564 | 1 | 65 | 2 | 1 | 3.5 | 0 | 0 | 0 | 0 | 172 | 1 | 0 | 0 |
| 18-24340 | 1 | 67 | 2 | 1 | 6.5 | 0 | 0 | 0 | 0 | 156 | 1 | 2 | 0 |
| 17-11155 | 1 | 70 | 2 | 1 | 7 | 1 | 0 | 0 | 0 | 126 | 1 | 2 | 0 |
| 18-34090 | 1 | 69 | 2 | 0 | 2 | 0 | 0 | 0 | 0 | 1724 | 0 | 3 | 0 |

(P Num., pathology number; Sex, as gender; A., age; V., Volume; L., maximum tumor long diameter; Diff., differentiation degree; TNM, TNM stage; LM., lymph node metastasis; DM., Distant Metastasis; OS., overall survival; SS., survival status; Score, IHC score; Group, stratification based on the Score. In the Gender column, "0" denotes a male patient, while "1" for a female patient. The Age column lists the actual age of the respective patient. The Volume is marked as "0" for tumors with a maximum long diameter less than 3 cm, and "1" for those with a diameter of 3 cm or more. The L. is given in centimeters (cm). Differentiation degree is categorized as "0" for poorly differentiated and "1" for well-differentiated pancreatic cancer. The TNM Stage column uses "0" for stages I and II, and "1" for stages III and IV. LM. is labeled as "0" when no lymph node metastasis is detected and "1" when one or more such metastases are present. DM. is noted as "0" for absence and "1" for the presence of distant metastasis to other organs. The OS. duration is calculated in days from the date of diagnosis to the date of death for deceased patients, or until the last follow-up for living patients. SS. is denoted as "0" for surviving patients and "1" for those who have deceased. The Score is derived from the IHC staining of LINC01605, where two pathologists independently rated the staining intensity and area, and the product of these ratings constitutes the final score. The Group categorizes cases into high and low LINC01605 expression groups based on the total score: "0" represents a score ≤4, classifying the sample as low expression, and "1" indicates a score >4, defining high expression.)

Supplementary Table 2. Primer sequences used in qRT-PCR assays.

| **Target gene** | **Forward sequence** | **Reverse sequence** |
| --- | --- | --- |
| *LINC01605* | CGTTACAAACAGCCGACCTT | CCAGGGAGGGACTCAAGAAT |
| *Lin28b* | CATCTCCATGATAAACCGAGAGG | GTTACCCGTATTGACTCAAGGC |
| *FDFT1* | CCACCCCGAAGAGTTCTACAA | TGCGACTGGTCTGATTGAGATA |
| *LDLR* | TCTGCAACATGGCTAGAGACT | TCCAAGCATTCGTTGGTCCC |
| *DHCR7* | GCTGCAAAATCGCAACCCAA | GCTCGCCAGTGAAAACCAGT |
| *HMGCS1* | GATGTGGGAATTGTTGCCCTT | ATTGTCTCTGTTCCAACTTCCAG |
| *HMGCR* | TGATTGACCTTTCCAGAGCAAG | CTAAAATTGCCATTCCACGAGC |
| *18S* | GGCCCTGTAATTGGAATGAGTC | CCAAGATCCAACTACGAGCTT |

Supplementary Table 3. Sequences used in gene knockdown assays.

| **Name** | **Sequence 5’ to 3’** |
| --- | --- |
| si-(Human)LINC01605-1 | CCUAGAACGGAGUCUUGAA |
| si-(Human)LINC01605-2  sh-(Human)LINC01605-1  sh-(Human)LINC01605-2  si-(Human)Lin28b-1  si-(Human)Lin28b-2 | CCAGUGAGAGAACAUACAA  CCUAGAACGGAGUCUUGAA  CCAGUGAGAGAACAUACAA  GGAUAUUCCAGUCGAUGUA  GGAGAUAGAUGCUACAACU |

Supplementary Table 4. The differentially expressed genes of Renji RNA-seq between tumor tissues and normal tissues.

| **Gene symbol** | **logFC** | **adj.P-Val** | **Gene symbol** | **logFC** | **adj.P-Val** |
| --- | --- | --- | --- | --- | --- |
| *AMY2B* | -6.68126 | 0.007187 | *HIST1H4H* | 2.354785 | 0.004288 |
| *GNMT* | -6.28667 | 0.005407 | *LINC00887* | 2.356993 | 0.007461 |
| *PM20D1* | -6.07552 | 0.002514 | *PHEX* | 2.357745 | 0.002545 |
| *MT1G* | -5.91889 | 0.000209 | *FAM72A* | 2.358839 | 0.002296 |
| *SPX* | -5.8539 | 0.001093 | *MELTF* | 2.361414 | 0.000531 |
| *IAPP* | -5.83459 | 0.000599 | *LINC02595* | 2.363103 | 0.006064 |
| *NPHS1* | -5.71844 | 0.001193 | *PARPBP* | 2.363382 | 0.005116 |
| *AF131216.3* | -5.70516 | 0.003765 | *SCD* | 2.368106 | 0.004972 |
| *SNTG2* | -5.64746 | 0.000119 | *SULF2* | 2.368615 | 0.006555 |
| *MT1H* | -5.56809 | 2.02E-05 | *FANCD2* | 2.370206 | 0.007827 |
| *TMED11P* | -5.42804 | 0.008232 | *AURKA* | 2.371108 | 0.00304 |
| *ARHGDIG* | -5.303 | 0.000708 | *OAS2* | 2.376762 | 0.003823 |
| *KCNK3* | -5.29663 | 0.001416 | *RIPPLY3* | 2.376778 | 0.002309 |
| *KIRREL2* | -5.26326 | 0.002587 | *LGALS9C* | 2.376944 | 0.005231 |
| *GUCA1C* | -5.16231 | 0.007524 | *STPG4* | 2.381545 | 0.006402 |
| *ATP4A* | -5.14041 | 0.00554 | *SIGLEC15* | 2.382653 | 0.00924 |
| *SCGN* | -5.10923 | 0.002681 | *SMCO2* | 2.38495 | 0.001946 |
| *TDH* | -5.0884 | 0.008067 | *TNFRSF21* | 2.386501 | 0.000142 |
| *CCKBR* | -4.96501 | 0.00175 | *AL590644.1* | 2.386648 | 0.006772 |
| *AC105272.1* | -4.92571 | 0.006686 | *GDA* | 2.38814 | 0.005247 |
| *P2RX1* | -4.9098 | 0.001426 | *STRIP2* | 2.391448 | 0.00704 |
| *BEX1* | -4.90313 | 0.002336 | *KIF24* | 2.395917 | 0.00319 |
| *MAT1A* | -4.81145 | 0.005394 | *HK2* | 2.407161 | 0.000806 |
| *FOSB* | -4.75362 | 0.000289 | *HPSE* | 2.407401 | 0.002204 |
| *CD300LG* | -4.6661 | 2.09E-06 | *MYB* | 2.415611 | 0.005434 |
| *AMY1B* | -4.65865 | 0.006494 | *AL137782.1* | 2.417971 | 0.009149 |
| *TEX11* | -4.64657 | 0.008312 | *APOL1* | 2.419792 | 0.006213 |
| *BRSK2* | -4.60566 | 0.001722 | *ENKUR* | 2.421576 | 0.006407 |
| *SPACA3* | -4.59115 | 0.009238 | *RACGAP1* | 2.422116 | 0.000323 |
| *PPY* | -4.5818 | 0.00982 | *IL7* | 2.422873 | 0.005592 |
| *HBB* | -4.57948 | 0.006769 | *AP1S3* | 2.430621 | 0.002022 |
| *KLB* | -4.48666 | 0.00166 | *LYPD1* | 2.439943 | 0.000859 |
| *RNU6-1022P* | -4.45686 | 0.009765 | *AL021807.1* | 2.440122 | 0.008112 |
| *CSDC2* | -4.44382 | 0.000731 | *LINC02041* | 2.441405 | 0.000694 |
| *KIAA1324* | -4.43151 | 0.001526 | *AC011352.3* | 2.443562 | 0.003012 |
| *TRHDE* | -4.36728 | 0.00846 | *PLK1* | 2.443872 | 0.000527 |
| *TMED6* | -4.35643 | 0.001377 | *AC099850.3* | 2.446743 | 0.001259 |
| *KIF1A* | -4.34203 | 0.00491 | *RASAL1* | 2.448113 | 0.002047 |
| *ANPEP* | -4.34099 | 0.00406 | *FOXD2* | 2.451097 | 0.001209 |
| *SLC8A2* | -4.33132 | 0.000725 | *BAIAP2L2* | 2.451341 | 0.000922 |
| *RNASE1* | -4.31081 | 0.001844 | *HIF1A-AS2* | 2.452325 | 0.003861 |
| *AMHR2* | -4.28006 | 0.003989 | *ADAMTSL5* | 2.45371 | 0.001153 |
| *LMO3* | -4.26599 | 0.000119 | *AC124947.2* | 2.465924 | 0.002079 |
| *HPCAL4* | -4.26456 | 0.000108 | *DCSTAMP* | 2.466486 | 0.006253 |
| *CA4* | -4.22679 | 0.003609 | *MBOAT2* | 2.467298 | 0.00166 |
| *EPHA8* | -4.2196 | 0.000335 | *DDIAS* | 2.472674 | 0.002454 |
| *PDK4* | -4.18598 | 8.78E-05 | *MAL2-AS1* | 2.475336 | 0.002422 |
| *PRODH2* | -4.17216 | 0.000209 | *AC108058.1* | 2.48098 | 0.004526 |
| *DPT* | -4.12181 | 0.002047 | *TGFBI* | 2.482416 | 0.001465 |
| *BTG2* | -4.10993 | 8.05E-05 | *S100A11* | 2.482459 | 0.000694 |
| *AOX1* | -4.10921 | 0.002454 | *MIR210HG* | 2.488882 | 0.000732 |
| *FAM167A-AS1* | -4.08133 | 0.006056 | *EME1* | 2.489015 | 0.002347 |
| *NGB* | -4.07132 | 0.007813 | *EPS8L1* | 2.489113 | 0.002903 |
| *VIPR2* | -4.05307 | 0.000139 | *WDR62* | 2.49028 | 0.00581 |
| *SLC1A2* | -4.04477 | 0.007802 | *LINC00920* | 2.493852 | 0.003611 |
| *SLC16A12* | -4.03239 | 0.004992 | *BCAN* | 2.493906 | 0.0017 |
| *ANKRD62* | -4.03099 | 0.009252 | *UBASH3B* | 2.497682 | 0.000708 |
| *NOS1* | -4.0199 | 0.00574 | *TMEM92-AS1* | 2.500802 | 0.001888 |
| *PEX5L* | -4.01986 | 0.000258 | *PIF1* | 2.51572 | 0.009721 |
| *ADIPOQ* | -3.99655 | 0.001648 | *TRIP13* | 2.51902 | 0.001281 |
| *LGALS2* | -3.96362 | 0.004366 | *PSORS1C1* | 2.520325 | 0.003831 |
| *LINC01251* | -3.92174 | 0.006656 | *ERFE* | 2.523284 | 0.002079 |
| *PAK3* | -3.91199 | 0.002374 | *FOXD2-AS1* | 2.525689 | 0.000272 |
| *C2CD4B* | -3.89534 | 0.001312 | *MLPH* | 2.527233 | 0.002847 |
| *PEBP4* | -3.88591 | 0.000289 | *BX470102.1* | 2.530135 | 0.003942 |
| *GAMT* | -3.84799 | 0.00175 | *SLC6A6* | 2.532014 | 0.008364 |
| *MYCL* | -3.84477 | 0.009655 | *HIST1H2BD* | 2.535125 | 0.002847 |
| *IGFN1* | -3.82912 | 0.002441 | *CIP2A* | 2.538184 | 0.003521 |
| *GSG1* | -3.79823 | 0.001439 | *PRR15* | 2.538587 | 0.002284 |
| *CAMK2N2* | -3.77908 | 0.000409 | *RIBC2* | 2.538639 | 0.005843 |
| *SPSB4* | -3.77262 | 0.002493 | *IL23A* | 2.54025 | 0.005329 |
| *KLF15* | -3.73623 | 0.000939 | *PKMYT1* | 2.543094 | 0.000641 |
| *AC123912.4* | -3.72876 | 0.009613 | *GINS4* | 2.547028 | 0.00306 |
| *TRBV29-1* | -3.72279 | 0.000305 | *ADGRG7* | 2.547573 | 0.002506 |
| *GPR150* | -3.71343 | 0.000793 | *FLRT3* | 2.550881 | 0.004097 |
| *ACADL* | -3.67248 | 0.003929 | *SDCBP2* | 2.551251 | 0.001484 |
| *PAIP2B* | -3.66089 | 0.003666 | *CABP4* | 2.551639 | 0.009965 |
| *ERO1B* | -3.65975 | 0.000216 | *CCNB1* | 2.55283 | 0.00155 |
| *KCNJ5* | -3.62044 | 0.002647 | *TRIM59* | 2.557777 | 0.004525 |
| *MYRIP* | -3.62003 | 0.000742 | *PPEF1* | 2.559745 | 0.001799 |
| *AC106795.2* | -3.5936 | 0.000209 | *KRT7-AS* | 2.56628 | 0.007767 |
| *FAM107A* | -3.58736 | 0.000197 | *RUNX2* | 2.566742 | 0.003324 |
| *GCAT* | -3.58209 | 0.003485 | *SIGLEC12* | 2.568282 | 0.008715 |
| *MT1XP1* | -3.55973 | 0.000717 | *OAS1* | 2.568539 | 0.000452 |
| *TMEM132C* | -3.55717 | 0.005423 | *SLC5A1* | 2.569123 | 0.001426 |
| *MTDHP3* | -3.55515 | 0.002256 | *HASPIN* | 2.571246 | 0.003439 |
| *SLC43A1* | -3.55447 | 0.003751 | *RAET1L* | 2.578152 | 0.009474 |
| *CNTFR* | -3.53584 | 0.000445 | *BCO1* | 2.585986 | 0.001893 |
| *LINC01754* | -3.53194 | 0.001411 | *TMEM154* | 2.591777 | 0.007902 |
| *SLITRK1* | -3.52902 | 0.000817 | *NCAPH* | 2.593783 | 0.001311 |
| *BANF2* | -3.52353 | 0.005188 | *TUBB3* | 2.601022 | 0.00342 |
| *AL590426.2* | -3.51302 | 0.001877 | *IGSF9* | 2.619458 | 0.005922 |
| *SARDH* | -3.50759 | 0.002336 | *ITGB4* | 2.619792 | 0.000601 |
| *MT1X* | -3.50718 | 0.000724 | *DLEU7* | 2.623223 | 0.00034 |
| *USP2* | -3.4958 | 4.57E-05 | *KIF23* | 2.626655 | 0.00082 |
| *AC005865.2* | -3.48943 | 0.004064 | *EVPL* | 2.627785 | 0.000806 |
| *ABAT* | -3.42612 | 0.00064 | *EDIL3* | 2.629949 | 0.008867 |
| *AL138930.1* | -3.41551 | 0.004646 | *KIF2C* | 2.633048 | 0.00064 |
| *HRASLS5* | -3.41315 | 0.005142 | *RNF223* | 2.6352 | 0.00971 |
| *DDC* | -3.41292 | 0.004325 | *HSD17B6* | 2.640097 | 0.003611 |
| *TCEAL2* | -3.40934 | 0.000784 | *MDFI* | 2.643343 | 0.004616 |
| *FABP4* | -3.39996 | 0.005542 | *PCLAF* | 2.644458 | 0.005525 |
| *FREM1* | -3.39265 | 0.002612 | *IL31RA* | 2.644719 | 0.009008 |
| *AC131097.2* | -3.37553 | 0.005741 | *RUNX1* | 2.645071 | 0.002644 |
| *DNASE1L3* | -3.37005 | 0.000533 | *FAM72D* | 2.653568 | 0.000748 |
| *RNF212* | -3.34661 | 0.001326 | *AC130456.3* | 2.656397 | 0.001449 |
| *SIGLEC11* | -3.30623 | 0.006659 | *PROC* | 2.662371 | 0.001941 |
| *LINC01354* | -3.28749 | 0.000185 | *FERMT1* | 2.662636 | 0.000209 |
| *MYOC* | -3.25983 | 0.000298 | *RNF183* | 2.66468 | 0.004821 |
| *TPST2* | -3.25604 | 0.007179 | *TMEM26* | 2.665599 | 0.007212 |
| *VWA5B2* | -3.24579 | 0.008521 | *AL157786.1* | 2.667292 | 0.000888 |
| *RARRES2* | -3.24144 | 0.004769 | *MACC1* | 2.67021 | 0.003295 |
| *FBXW12* | -3.23455 | 0.00941 | *CDCA3* | 2.674666 | 0.004435 |
| *RAB26* | -3.21138 | 0.000374 | *HIST1H2AI* | 2.675025 | 0.000472 |
| *HMGB3P27* | -3.20541 | 0.005113 | *NEIL3* | 2.682883 | 0.000291 |
| *AMER3* | -3.1927 | 0.001539 | *KIF15* | 2.683054 | 0.002296 |
| *TMEM225* | -3.17639 | 0.007571 | *ANO1* | 2.684446 | 0.000618 |
| *MYMX* | -3.17176 | 0.008102 | *AL021392.1* | 2.686521 | 0.004992 |
| *FITM1* | -3.17155 | 0.000708 | *GLI2* | 2.689385 | 0.0096 |
| *RIC3* | -3.16735 | 0.006131 | *PITX2* | 2.692694 | 0.001722 |
| *FLT3* | -3.14129 | 0.001107 | *RASSF10* | 2.693399 | 0.007028 |
| *GSTA9P* | -3.12676 | 0.003485 | *PLAU* | 2.695409 | 0.009528 |
| *NUPR1* | -3.12505 | 0.002712 | *HOXB7* | 2.695861 | 0.005915 |
| *ACSM6* | -3.10901 | 0.001402 | *CENPU* | 2.695939 | 0.000258 |
| *RADIL* | -3.09652 | 0.000933 | *AC013268.3* | 2.698431 | 0.000562 |
| *C2orf27B* | -3.09077 | 0.002823 | *LINC00460* | 2.698897 | 0.003994 |
| *AL109910.2* | -3.08488 | 0.006631 | *CDCA8* | 2.699719 | 0.001193 |
| *C10orf82* | -3.07988 | 0.003341 | *HOXB3* | 2.704223 | 0.001607 |
| *CXCL12* | -3.07704 | 0.004815 | *MAL2* | 2.707436 | 0.001135 |
| *MT1F* | -3.04442 | 0.000838 | *ERCC6L* | 2.707712 | 0.003714 |
| *C16orf89* | -3.0429 | 0.00276 | *MAD2L1* | 2.70969 | 0.000435 |
| *AC021146.11* | -3.04287 | 0.00314 | *DIAPH3* | 2.715101 | 7.65E-05 |
| *CCDC141* | -3.0425 | 0.009886 | *ADAMTS12* | 2.715247 | 0.005104 |
| *TENT5C* | -3.02364 | 0.000445 | *GINS1* | 2.719346 | 0.000209 |
| *PTGER4* | -3.01947 | 0.000489 | *FXYD3* | 2.721944 | 0.003133 |
| *RXRG* | -2.9838 | 0.008954 | *SGO1* | 2.727493 | 0.003823 |
| *APLP1* | -2.97831 | 0.001944 | *TEDC2* | 2.730703 | 0.001087 |
| *MYT1L* | -2.97162 | 0.005874 | *CENPM* | 2.740953 | 0.003133 |
| *ECHDC3* | -2.96527 | 0.002197 | *OSBPL3* | 2.748769 | 0.000305 |
| *NAT8L* | -2.9548 | 0.008004 | *SPAG5* | 2.762925 | 0.001313 |
| *LINC01625* | -2.94368 | 0.000432 | *AC025539.1* | 2.767505 | 0.000694 |
| *TECPR1* | -2.93978 | 0.002168 | *ITGB6* | 2.768148 | 0.005866 |
| *TSPAN7* | -2.93853 | 0.003871 | *S100A6* | 2.769141 | 0.002925 |
| *XKR4* | -2.91601 | 0.001107 | *NRP2* | 2.771526 | 0.004324 |
| *TRBV11-2* | -2.909 | 0.000363 | *ADAM28* | 2.772147 | 0.001648 |
| *LINC01529* | -2.9019 | 0.001069 | *NHS* | 2.772911 | 0.003494 |
| *ECE2* | -2.89759 | 0.00514 | *NECTIN4* | 2.777801 | 0.00146 |
| *UNC79* | -2.88597 | 0.001692 | *IQANK1* | 2.779743 | 0.003396 |
| *MT1M* | -2.88213 | 0.000742 | *PLEKHN1* | 2.780099 | 0.000223 |
| *MAPK8IP1* | -2.88116 | 0.000323 | *AC012531.1* | 2.781666 | 0.008605 |
| *AC116614.1* | -2.88031 | 0.000445 | *ZIC5* | 2.781776 | 0.007028 |
| *SORCS1* | -2.8587 | 0.003849 | *PLEK2* | 2.78662 | 0.005109 |
| *CERS4* | -2.85803 | 0.00138 | *RAD51* | 2.787079 | 0.004388 |
| *NUCB2* | -2.85476 | 0.002245 | *CD109* | 2.790155 | 0.005547 |
| *CCDC110* | -2.85027 | 0.003743 | *COL7A1* | 2.797492 | 0.004437 |
| *FGF10* | -2.83996 | 0.006864 | *SLFN13* | 2.7977 | 0.000259 |
| *LCN10* | -2.83866 | 0.00064 | *CKS2* | 2.80025 | 0.003164 |
| *LINC01238* | -2.8347 | 0.000896 | *BRIP1* | 2.808359 | 0.004041 |
| *RGN* | -2.83237 | 0.003959 | *CDCA5* | 2.814533 | 0.000724 |
| *FFAR1* | -2.82565 | 0.009901 | *TPSP2* | 2.816397 | 0.001196 |
| *SDHDP1* | -2.82025 | 0.004343 | *VWA3B* | 2.81745 | 0.000677 |
| *EPHX2* | -2.81233 | 0.001107 | *SLC17A9* | 2.822864 | 0.000585 |
| *MTUS2* | -2.81048 | 0.003765 | *E2F1* | 2.823909 | 0.000298 |
| *NRTN* | -2.8053 | 0.001946 | *SKA1* | 2.833125 | 0.001935 |
| *ANKRD53* | -2.80318 | 0.000351 | *RAD51AP1* | 2.837292 | 0.000677 |
| *CBFA2T3* | -2.80034 | 0.000725 | *SERTAD4* | 2.838603 | 0.002847 |
| *SEMA6D* | -2.78512 | 0.000782 | *KIF26B* | 2.851706 | 0.005923 |
| *C14orf180* | -2.78471 | 0.002031 | *PPFIA4* | 2.854224 | 0.006008 |
| *BEX5* | -2.77852 | 0.001093 | *TMC7* | 2.859135 | 0.003853 |
| *BNIP3* | -2.77648 | 0.006946 | *CCNB2* | 2.859414 | 0.000272 |
| *NRCAM* | -2.77361 | 0.005202 | *RHBDL2* | 2.859792 | 0.000708 |
| *OGDHL* | -2.77103 | 0.004321 | *FNDC11* | 2.861973 | 0.002047 |
| *BACE1* | -2.76404 | 0.003573 | *IRX5* | 2.874442 | 0.005424 |
| *NKX6-1* | -2.75689 | 0.007212 | *ESPN* | 2.876821 | 0.003994 |
| *CHRNE* | -2.74519 | 0.000742 | *TRIM10* | 2.88617 | 0.000298 |
| *TPO* | -2.73891 | 0.002309 | *NT5E* | 2.886711 | 0.003049 |
| *TRBV5-4* | -2.73759 | 0.004962 | *SMPX* | 2.887138 | 0.0026 |
| *CCDC69* | -2.7255 | 0.000196 | *PLK4* | 2.891534 | 0.000205 |
| *PTPRN2* | -2.71867 | 0.001956 | *TRPM8* | 2.89656 | 0.005818 |
| *NR4A1* | -2.71281 | 0.000534 | *LINC02535* | 2.901897 | 0.001729 |
| *PLD5* | -2.6979 | 0.000605 | *FAM83B* | 2.903453 | 0.005329 |
| *PDCD4* | -2.69674 | 0.004027 | *HOXB5* | 2.9053 | 0.003751 |
| *AP000785.2* | -2.6801 | 0.001859 | *AK4* | 2.913459 | 0.000863 |
| *RYR2* | -2.66995 | 0.000185 | *PRR11* | 2.91581 | 0.002074 |
| *TUBB4A* | -2.66765 | 0.00054 | *LRP8* | 2.917279 | 0.000838 |
| *AGTR2* | -2.65508 | 0.000657 | *KRT7* | 2.930667 | 0.002853 |
| *AC092427.1* | -2.65504 | 0.003121 | *SEMA7A* | 2.934092 | 0.006496 |
| *NANOGP1* | -2.64827 | 0.001971 | *MET* | 2.938589 | 0.000227 |
| *ANGPTL1* | -2.63389 | 0.000209 | *SLC2A1* | 2.941501 | 0.006346 |
| *ADAMTS16* | -2.62631 | 0.001408 | *AC112777.1* | 2.943876 | 0.001425 |
| *AC129507.1* | -2.62504 | 0.007274 | *CDKN2B* | 2.967289 | 0.003765 |
| *PACSIN1* | -2.61939 | 0.005302 | *KLF5* | 2.984833 | 0.000108 |
| *CDH23* | -2.61793 | 0.000235 | *CDC25C* | 2.985745 | 0.004437 |
| *LIFR* | -2.61195 | 0.000203 | *DUOXA1* | 2.988428 | 0.002334 |
| *ARL5C* | -2.61089 | 0.006745 | *TMEM171* | 2.992005 | 0.000305 |
| *GAS1RR* | -2.60928 | 0.001311 | *HTR1D* | 2.993448 | 0.002567 |
| *RAB39B* | -2.60635 | 0.001941 | *PTPRR* | 2.998366 | 0.002118 |
| *SYBU* | -2.5925 | 0.007596 | *MTFR2* | 2.999627 | 0.000562 |
| *TCEA3* | -2.58943 | 0.006211 | *HOXB-AS3* | 3.002107 | 0.003714 |
| *PARD6A* | -2.58911 | 0.001844 | *NUSAP1* | 3.002139 | 0.000161 |
| *POMC* | -2.58664 | 0.003368 | *ARNTL2* | 3.003706 | 0.000506 |
| *SFTPD* | -2.58268 | 0.001166 | *PCDH7* | 3.00504 | 0.007891 |
| *IGSF11* | -2.58171 | 0.000865 | *AC140479.2* | 3.008823 | 0.000708 |
| *CECR2* | -2.55747 | 0.000972 | *TNFSF11* | 3.009405 | 0.006671 |
| *AC006033.1* | -2.55595 | 0.002697 | *TPRG1* | 3.015809 | 0.00795 |
| *AC106795.3* | -2.55081 | 0.000259 | *CDKN3* | 3.015995 | 0.002339 |
| *DTNA* | -2.54938 | 0.000724 | *SH3TC2* | 3.017204 | 0.000635 |
| *TRBV6-1* | -2.54484 | 0.000152 | *TNFSF15* | 3.034679 | 0.001424 |
| *FGF12* | -2.54224 | 0.005813 | *CLSPN* | 3.04445 | 0.002455 |
| *PHYHD1* | -2.53862 | 0.000907 | *AL590666.2* | 3.052904 | 0.001326 |
| *TRARG1* | -2.53824 | 0.008853 | *HOPX* | 3.0532 | 0.008476 |
| *NMUR1* | -2.53691 | 0.00775 | *AC130456.2* | 3.058062 | 0.005201 |
| *KIF26A* | -2.53488 | 0.005928 | *ACTBL2* | 3.062317 | 0.00405 |
| *KCNK10* | -2.52216 | 0.001831 | *IBSP* | 3.065621 | 0.000488 |
| *EPHX1* | -2.51366 | 0.000863 | *LINC00857* | 3.066293 | 0.000289 |
| *DIRAS3* | -2.5096 | 0.001424 | *FOXM1* | 3.066421 | 0.000502 |
| *CELF4* | -2.504 | 0.00638 | *AC006262.1* | 3.079156 | 0.000806 |
| *TMEM63C* | -2.488 | 0.008737 | *ECT2* | 3.080399 | 0.000865 |
| *TRBV6-5* | -2.48469 | 0.001283 | *ASF1B* | 3.086957 | 0.000914 |
| *AC092535.1* | -2.47985 | 0.00402 | *DMBX1* | 3.096499 | 0.001807 |
| *BEGAIN* | -2.47847 | 0.009937 | *SPACA4* | 3.097707 | 0.006671 |
| *SSR4* | -2.47641 | 0.003765 | *BEAN1* | 3.100263 | 0.001107 |
| *CYS1* | -2.47082 | 0.006772 | *EFNA2* | 3.113711 | 0.007524 |
| *LINGO4* | -2.46675 | 0.000305 | *CDC45* | 3.114214 | 0.00174 |
| *FAM129A* | -2.45847 | 0.000669 | *DTL* | 3.115479 | 0.000311 |
| *KCNJ11* | -2.45166 | 0.0025 | *MROH6* | 3.117236 | 0.001497 |
| *ANKRD20A8P* | -2.44535 | 0.006772 | *CENPA* | 3.117601 | 0.003553 |
| *SLC25A34* | -2.43711 | 0.000139 | *DNAH3* | 3.123348 | 0.003765 |
| *TRIM73* | -2.43392 | 0.002635 | *ORC6* | 3.146277 | 0.000398 |
| *NEXMIF* | -2.43016 | 0.001186 | *NQO1* | 3.146331 | 0.000221 |
| *SEC11C* | -2.42979 | 0.000785 | *KPNA7* | 3.151996 | 0.000209 |
| *FKBP11* | -2.42522 | 0.000899 | *CACNG8* | 3.153724 | 0.00918 |
| *FAM217A* | -2.42514 | 0.004594 | *HIST1H2AG* | 3.155395 | 0.001263 |
| *SYP* | -2.42377 | 0.003626 | *HIST1H2BJ* | 3.15809 | 0.005728 |
| *AC016924.1* | -2.42213 | 0.000272 | *PMAIP1* | 3.168272 | 0.001995 |
| *FAM153C* | -2.41802 | 0.003823 | *CELSR1* | 3.170011 | 0.000152 |
| *PLTP* | -2.41731 | 0.000278 | *HOXC6* | 3.17908 | 0.003848 |
| *PEX6* | -2.40571 | 0.006434 | *XRCC2* | 3.180131 | 0.000853 |
| *FAM110D* | -2.40444 | 0.002765 | *STIL* | 3.180392 | 0.00047 |
| *CYP4F32P* | -2.39555 | 0.001406 | *ESPL1* | 3.184565 | 0.001018 |
| *LINC00940* | -2.39222 | 0.007587 | *SHCBP1* | 3.185967 | 0.002656 |
| *XBP1* | -2.39039 | 0.008981 | *CENPK* | 3.188698 | 0.001373 |
| *AC010136.1* | -2.38813 | 0.001183 | *CCNA2* | 3.192128 | 0.000227 |
| *NTRK2* | -2.38552 | 0.00704 | *MST1R* | 3.193368 | 0.000305 |
| *AC002451.1* | -2.38008 | 0.003439 | *TMEM45B* | 3.198851 | 0.000251 |
| *REEP1* | -2.37725 | 0.002554 | *SLC4A11* | 3.199651 | 0.002447 |
| *CD36* | -2.36995 | 0.002644 | *KIFC1* | 3.214994 | 0.000276 |
| *AP000757.1* | -2.35956 | 0.002928 | *SAPCD2* | 3.220163 | 0.001209 |
| *DPPA2P4* | -2.35443 | 0.005032 | *TTK* | 3.222015 | 0.000313 |
| *TRBV10-2* | -2.34693 | 0.003739 | *HOXB6* | 3.222482 | 0.004325 |
| *KCNJ8* | -2.34623 | 0.003823 | *LINC02562* | 3.225188 | 0.002644 |
| *AC106047.1* | -2.34374 | 0.003266 | *ATG9B* | 3.226246 | 0.005116 |
| *C5* | -2.34307 | 0.004874 | *MUC13* | 3.227472 | 0.00845 |
| *PLIN4* | -2.34227 | 0.000374 | *CLIC3* | 3.237848 | 0.002814 |
| *ATF3* | -2.34123 | 0.001099 | *KCP* | 3.237954 | 0.005834 |
| *RPH3AL* | -2.3271 | 0.000376 | *MMP10* | 3.23821 | 0.005218 |
| *AL121929.2* | -2.32256 | 0.00034 | *AMN* | 3.249117 | 0.008879 |
| *RGMA* | -2.32171 | 0.000605 | *LINP1* | 3.253623 | 0.00404 |
| *AQP1* | -2.31853 | 0.004358 | *WFDC2* | 3.253987 | 0.007713 |
| *PGM5P4* | -2.31019 | 0.005937 | *LINC02081* | 3.254118 | 0.000291 |
| *AL355877.1* | -2.30714 | 0.001103 | *NTM* | 3.264765 | 0.003121 |
| *ASTN2* | -2.30207 | 0.000185 | *TNNI2* | 3.266749 | 0.008373 |
| *ADAM7* | -2.29889 | 0.009997 | *SHISA2* | 3.271926 | 0.005345 |
| *TRBV6-7* | -2.28334 | 0.004654 | *PHLDA2* | 3.273813 | 0.002467 |
| *SYT9* | -2.26814 | 0.008738 | *ZNF860* | 3.275016 | 0.000708 |
| *ATP2A3* | -2.26775 | 0.00304 | *CORO2A* | 3.275236 | 0.001209 |
| *SCAMP5* | -2.26464 | 0.000466 | *CTSV* | 3.283904 | 0.005426 |
| *PLEKHH3* | -2.25994 | 0.003929 | *TGM2* | 3.302152 | 0.00081 |
| *VLDLR-AS1* | -2.25185 | 0.000349 | *CHST6* | 3.303037 | 0.006455 |
| *ECI2* | -2.25079 | 0.002767 | *THBS2* | 3.307487 | 0.007891 |
| *GREB1* | -2.24767 | 0.00173 | *CENPI* | 3.308356 | 0.000445 |
| *ADD2* | -2.24599 | 0.001356 | *KISS1* | 3.325327 | 0.007827 |
| *ALDH1A1* | -2.23982 | 5.79E-05 | *CDH3* | 3.327893 | 0.001438 |
| *IP6K3* | -2.23413 | 0.002166 | *CENPE* | 3.328861 | 0.002079 |
| *MSI1* | -2.22981 | 0.004449 | *PTTG1* | 3.330737 | 0.001439 |
| *LINC01352* | -2.22778 | 0.002847 | *CPNE7* | 3.338082 | 0.002031 |
| *MRO* | -2.22338 | 0.000951 | *MCM10* | 3.339303 | 0.000953 |
| *MKNK1* | -2.22012 | 0.002191 | *KYNU* | 3.345784 | 0.002572 |
| *TRBV12-1* | -2.21997 | 0.009833 | *CST4* | 3.36229 | 0.005032 |
| *MYH6* | -2.2184 | 0.000888 | *NMRAL2P* | 3.36463 | 0.000597 |
| *HAAO* | -2.21835 | 0.000357 | *RTKN2* | 3.381546 | 0.001745 |
| *APOBEC2* | -2.21756 | 0.00894 | *GPR35* | 3.3849 | 0.000708 |
| *AL122008.3* | -2.21454 | 0.00614 | *CYP2C18* | 3.397927 | 0.006068 |
| *RGS2* | -2.2126 | 0.003985 | *CDCA2* | 3.403399 | 0.000298 |
| *CASKIN1* | -2.20617 | 0.003994 | *ABCA12* | 3.403688 | 0.003671 |
| *OR6M1* | -2.20304 | 0.00504 | *SH3D21* | 3.431703 | 0.000677 |
| *CYB5A* | -2.20164 | 0.002572 | *AURKB* | 3.441361 | 0.000977 |
| *LINC00339* | -2.19386 | 0.001943 | *TRIM31* | 3.44179 | 0.003609 |
| *ACAT1* | -2.19241 | 0.000509 | *EPHX4* | 3.446372 | 0.000452 |
| *ERC2* | -2.18906 | 0.004772 | *ITGA2* | 3.447629 | 0.000305 |
| *TRBV7-6* | -2.18903 | 0.000259 | *CST6* | 3.450412 | 0.001424 |
| *TRIM74* | -2.18411 | 0.008991 | *CALB2* | 3.453674 | 0.009897 |
| *AC105277.1* | -2.18346 | 0.002572 | *KIF18A* | 3.45644 | 0.000602 |
| *DHRS12* | -2.17932 | 0.000521 | *CTHRC1* | 3.458257 | 0.00603 |
| *SLC25A25* | -2.17804 | 0.001941 | *TNFRSF11B* | 3.466441 | 0.002572 |
| *LINC02447* | -2.17727 | 0.000975 | *RRM2* | 3.477329 | 0.000445 |
| *LCN6* | -2.17684 | 5.78E-05 | *PNCK* | 3.479924 | 0.003989 |
| *C17orf107* | -2.17447 | 0.004736 | *SLC16A3* | 3.49402 | 0.000266 |
| *SIDT2* | -2.16911 | 0.000742 | *TMEM105* | 3.517418 | 0.000445 |
| *CTH* | -2.16864 | 0.004128 | *COL22A1* | 3.532217 | 0.002814 |
| *RASIP1* | -2.16707 | 8.78E-05 | *HMMR* | 3.540598 | 0.00034 |
| *GSTM2* | -2.1665 | 0.002134 | *EFNA5* | 3.555798 | 0.001722 |
| *C16orf96* | -2.16522 | 0.001402 | *CDK1* | 3.559607 | 0.001371 |
| *FO681548.1* | -2.15777 | 0.00166 | *RFLNA* | 3.57162 | 0.000305 |
| *F8* | -2.15754 | 0.003313 | *BNIP3P4* | 3.5718 | 0.007102 |
| *PRLHR* | -2.15751 | 0.007891 | *GTSE1* | 3.581835 | 0.000296 |
| *SLC25A45* | -2.15044 | 0.001163 | *CA9* | 3.581909 | 0.009154 |
| *SYPL2* | -2.14456 | 0.002166 | *CLRN3* | 3.58511 | 0.002836 |
| *HFM1* | -2.13445 | 0.005525 | *PADI3* | 3.590285 | 0.009886 |
| *PABPC4* | -2.13263 | 0.000806 | *CYP2S1* | 3.593833 | 0.004027 |
| *AC073316.3* | -2.13134 | 0.00431 | *FRMD5* | 3.599072 | 0.001107 |
| *RPL3* | -2.12924 | 0.000272 | *HOXA13* | 3.603595 | 0.008442 |
| *FOXP2* | -2.11171 | 0.000259 | *DEPDC1* | 3.608217 | 0.001449 |
| *TMEM131L* | -2.11036 | 0.000852 | *DEPDC1B* | 3.608394 | 0.000272 |
| *DHRS4L1* | -2.10951 | 0.003468 | *HES2* | 3.610259 | 0.002065 |
| *GFI1* | -2.10616 | 0.002339 | *LINC00941* | 3.611537 | 0.000782 |
| *AP3B2* | -2.10505 | 0.00806 | *FER1L4* | 3.614956 | 0.000276 |
| *TSPAN33* | -2.09014 | 0.004246 | *PNPLA3* | 3.615787 | 0.000454 |
| *FLRT2* | -2.08941 | 0.007911 | *CRYBG2* | 3.622007 | 0.002506 |
| *AC123768.3* | -2.08919 | 0.000452 | *EXO1* | 3.624671 | 0.000266 |
| *GAS2* | -2.08027 | 0.003164 | *OXTR* | 3.628048 | 0.003751 |
| *KHK* | -2.07713 | 0.008013 | *MAB21L4* | 3.641223 | 0.005016 |
| *SLC39A14* | -2.07546 | 0.00846 | *GUCY1B2* | 3.64555 | 0.002309 |
| *CRAT* | -2.07514 | 0.007001 | *GSDMC* | 3.653691 | 0.000599 |
| *FGF14-AS2* | -2.0688 | 0.008897 | *PIMREG* | 3.655482 | 0.000196 |
| *DUSP1* | -2.06722 | 0.000305 | *DNAH11* | 3.663987 | 0.007323 |
| *PLCE1* | -2.0628 | 0.000754 | *WTAPP1* | 3.664713 | 0.001995 |
| *JUN* | -2.06217 | 0.000101 | *LEF1* | 3.667583 | 0.007497 |
| *SMIM10L2A* | -2.05265 | 0.002399 | *B3GNT3* | 3.681524 | 0.000253 |
| *NOP53* | -2.05082 | 0.000289 | *GPX2* | 3.682241 | 0.000221 |
| *ATOH8* | -2.05036 | 0.009532 | *ZG16B* | 3.695159 | 0.003164 |
| *AL137026.1* | -2.04801 | 0.004727 | *MANCR* | 3.698651 | 0.000724 |
| *SLC39A8* | -2.04701 | 0.009952 | *FAM83E* | 3.698655 | 0.003111 |
| *TF* | -2.04226 | 0.005124 | *AHNAK2* | 3.700389 | 0.001939 |
| *LINC01816* | -2.03808 | 0.001463 | *IGFBP3* | 3.700998 | 0.000464 |
| *PKHD1L1* | -2.03456 | 0.006505 | *SYT12* | 3.703035 | 0.001573 |
| *EGR1* | -2.02932 | 0.00292 | *E2F7* | 3.70589 | 0.000341 |
| *DCT* | -2.02084 | 0.006402 | *SYT8* | 3.706013 | 0.002097 |
| *ERN1* | -2.02062 | 0.002971 | *KRT19* | 3.708363 | 0.001949 |
| *AC126768.2* | -2.01453 | 0.009159 | *CEMIP* | 3.712149 | 0.000381 |
| *LRRC2* | -2.01372 | 0.003337 | *UBE2T* | 3.713341 | 0.000276 |
| *AL035706.1* | -2.01335 | 0.007001 | *BIRC5* | 3.72368 | 0.001156 |
| *AC139712.1* | -2.01181 | 0.006488 | *ZIC2* | 3.746659 | 0.002619 |
| *SKIDA1* | -2.00455 | 0.006686 | *EGLN3* | 3.752412 | 0.000185 |
| *AL049775.1* | -2.0031 | 0.006525 | *SLPI* | 3.757749 | 0.001294 |
| *JUND* | -2.00109 | 0.000838 | *STYK1* | 3.758863 | 0.005012 |
| *SPRY4-AS1* | 2.002366 | 0.006064 | *SULF1* | 3.766075 | 0.001949 |
| *CAPN5* | 2.004035 | 0.003994 | *NCAPG* | 3.768181 | 0.000161 |
| *CIT* | 2.004916 | 0.008457 | *HS3ST1* | 3.801619 | 0.000363 |
| *VDR* | 2.005704 | 0.009128 | *MISP* | 3.807115 | 2.21E-05 |
| *LMNB1* | 2.006958 | 0.005432 | *LINC02577* | 3.818176 | 0.008442 |
| *OAS3* | 2.013657 | 0.003917 | *PTK6* | 3.822721 | 0.000209 |
| *PLA2R1* | 2.014188 | 0.007713 | *BUB1B* | 3.843194 | 0.000197 |
| *C19orf33* | 2.014454 | 0.005482 | *MELK* | 3.845649 | 0.000209 |
| *ARHGAP42* | 2.019017 | 0.006525 | *KRT23* | 3.847706 | 0.00124 |
| *SPAG1* | 2.020148 | 0.000469 | *TICRR* | 3.852101 | 0.000381 |
| *GALE* | 2.021068 | 0.003959 | *CEACAM1* | 3.852966 | 5.78E-05 |
| *MGLL* | 2.021405 | 0.002567 | *ADAMTS14* | 3.881692 | 0.001196 |
| *TGFB2* | 2.021567 | 0.002644 | *VCAN* | 3.886839 | 0.002245 |
| *INPP4B* | 2.022044 | 0.003841 | *E2F8* | 3.896361 | 0.000265 |
| *AL606489.1* | 2.030004 | 0.000708 | *CKAP2L* | 3.902328 | 0.000289 |
| *PCDHB10* | 2.032194 | 0.004325 | *LEMD1* | 3.903609 | 0.001402 |
| *MYOF* | 2.037038 | 0.006064 | *ALDH3B2* | 3.91273 | 0.001438 |
| *CYP51A1P2* | 2.03757 | 0.005589 | *MMP1* | 3.957963 | 0.006128 |
| *AC009237.15* | 2.03965 | 0.002493 | *POLQ* | 3.962091 | 0.000445 |
| *AC023908.3* | 2.043055 | 0.003063 | *ESM1* | 3.966625 | 0.000265 |
| *AC233280.2* | 2.044204 | 0.001332 | *KIF4A* | 3.973308 | 0.000216 |
| *TRIM46* | 2.045849 | 0.008527 | *CENPF* | 3.980353 | 0.000172 |
| *PTPRH* | 2.046107 | 0.002055 | *KNL1* | 4.000257 | 0.00041 |
| *HOXB-AS1* | 2.049782 | 0.004546 | *EDAR* | 4.006412 | 0.000396 |
| *CDC6* | 2.054385 | 0.009714 | *GJB4* | 4.034326 | 0.005083 |
| *ETV1* | 2.056162 | 0.001888 | *NEK2* | 4.038568 | 0.000353 |
| *UCN2* | 2.0566 | 0.008071 | *SULT2B1* | 4.040809 | 0.000472 |
| *TM6SF2* | 2.058383 | 0.00298 | *KIF18B* | 4.043766 | 0.000521 |
| *EFNB2* | 2.05923 | 0.00276 | *TCN1* | 4.046632 | 0.001439 |
| *NDP* | 2.069047 | 0.00379 | *FAM83D* | 4.047583 | 0.000314 |
| *AC004231.1* | 2.070759 | 0.001402 | *TROAP* | 4.047831 | 0.000341 |
| *AC114488.1* | 2.078153 | 0.004546 | *FN1* | 4.052372 | 0.001193 |
| *LY75* | 2.090198 | 0.001915 | *FIBCD1* | 4.059717 | 0.000209 |
| *PSMD10P2* | 2.093272 | 0.004335 | *INHBA* | 4.079727 | 0.002579 |
| *U62317.1* | 2.09398 | 0.008169 | *SOX11* | 4.08488 | 0.000259 |
| *JPT1* | 2.096969 | 0.007617 | *WNT10A* | 4.093912 | 0.001153 |
| *CFAP54* | 2.09725 | 0.008232 | *KIF14* | 4.117036 | 0.000539 |
| *SAMD9* | 2.097424 | 0.003307 | *CEP55* | 4.121986 | 0.000268 |
| *KLHL2P1* | 2.100514 | 0.001426 | *SKA3* | 4.128834 | 0.000298 |
| *AC012613.2* | 2.101186 | 0.000841 | *NUF2* | 4.13814 | 0.000258 |
| *SLC52A3* | 2.107093 | 0.000142 | *KCNN4* | 4.15048 | 0.00064 |
| *AC104966.1* | 2.117463 | 0.005113 | *POSTN* | 4.153615 | 0.001129 |
| *BRCA2* | 2.120188 | 0.003841 | *LAMB3* | 4.15867 | 0.000209 |
| *PLA2G4D* | 2.120791 | 0.00462 | *LINC01614* | 4.173142 | 0.001455 |
| *AC100861.1* | 2.12099 | 0.006111 | *TSPAN1* | 4.177652 | 0.004517 |
| *SORD2P* | 2.123666 | 0.003635 | *C5orf46* | 4.186576 | 0.000119 |
| *DCAF4L1* | 2.123852 | 0.004248 | *UBE2C* | 4.197656 | 0.000785 |
| *AP005230.1* | 2.125387 | 0.007571 | *GJB2* | 4.215224 | 0.000276 |
| *BICD1* | 2.127753 | 0.000522 | *AOC1* | 4.217694 | 0.000931 |
| *TBX4* | 2.128666 | 0.000538 | *CDC20* | 4.243182 | 0.000635 |
| *SYTL2* | 2.129032 | 0.00248 | *FAM111B* | 4.248248 | 0.000381 |
| *CMTM1* | 2.140409 | 0.001768 | *WNT7B* | 4.260963 | 0.000708 |
| *ORC1* | 2.145896 | 0.000995 | *KRT17* | 4.288699 | 0.0039 |
| *IL1RAP* | 2.15499 | 0.000731 | *TPX2* | 4.306101 | 0.000108 |
| *AL049555.1* | 2.159192 | 0.001935 | *SHH* | 4.322137 | 0.000452 |
| *DNAAF3* | 2.163426 | 0.004268 | *SDR16C5* | 4.33632 | 0.004736 |
| *AC091057.4* | 2.16407 | 0.005186 | *TFAP2A* | 4.343479 | 0.000142 |
| *DUSP5P1* | 2.165123 | 0.001935 | *HAPLN1* | 4.348827 | 0.000899 |
| *C17orf53* | 2.168121 | 0.002759 | *AL359955.1* | 4.362885 | 0.001103 |
| *LINC01605* | 2.171815 | 0.009594 | *CAPN8* | 4.372994 | 0.001439 |
| *DGKH* | 2.172099 | 0.00846 | *BUB1* | 4.38874 | 0.000158 |
| *ABLIM3* | 2.177065 | 0.004781 | *TOP2A* | 4.405753 | 0.000289 |
| *NDC80* | 2.178824 | 0.00865 | *GCNT3* | 4.411309 | 0.003861 |
| *AC068631.1* | 2.180221 | 0.000108 | *KIF20A* | 4.443512 | 8.78E-05 |
| *SPRED3* | 2.181391 | 0.007257 | *COL11A1* | 4.449095 | 0.005301 |
| *LINC02154* | 2.18413 | 0.004972 | *IGFL2-AS1* | 4.458005 | 0.005052 |
| *CLDN23* | 2.196734 | 0.004763 | *MMP11* | 4.486243 | 0.000599 |
| *KIF11* | 2.197287 | 0.000899 | *GPR87* | 4.497366 | 0.005116 |
| *AC009121.1* | 2.197488 | 0.000874 | *DLGAP5* | 4.51274 | 0.000129 |
| *C3orf67* | 2.199725 | 0.002582 | *SPOCD1* | 4.514174 | 0.000216 |
| *AC009065.5* | 2.206282 | 0.007179 | *MKI67* | 4.519164 | 0.000164 |
| *FOXQ1* | 2.211447 | 0.008228 | *EPS8L3* | 4.554696 | 0.000838 |
| *SPAG17* | 2.211617 | 0.000259 | *LCN2* | 4.560007 | 0.001332 |
| *C6orf223* | 2.213099 | 0.008608 | *CEACAM6* | 4.561023 | 0.006444 |
| *CAMK2N1* | 2.221769 | 0.002302 | *AL365181.3* | 4.585922 | 0.000319 |
| *CARD11* | 2.223998 | 0.000964 | *LAMC2* | 4.596536 | 0.000216 |
| *DDX11-AS1* | 2.226172 | 0.002438 | *AL365181.2* | 4.610057 | 0.001184 |
| *MARCKSL1* | 2.226184 | 0.000499 | *MUC4* | 4.634769 | 0.008004 |
| *SLC7A4* | 2.227474 | 0.006631 | *TRIM15* | 4.647753 | 5.78E-05 |
| *MTMR11* | 2.229001 | 0.003113 | *SERPINB5* | 4.692028 | 0.003994 |
| *SLC22A18AS* | 2.235007 | 0.008678 | *STRA6* | 4.770827 | 0.000276 |
| *AC108463.3* | 2.241723 | 0.006048 | *TMC5* | 4.848143 | 0.000272 |
| *PACERR* | 2.243959 | 0.002454 | *MMP13* | 4.854159 | 0.000445 |
| *PLS1* | 2.244837 | 0.000724 | *CCL20* | 4.869267 | 0.001483 |
| *IER5L* | 2.248299 | 0.008769 | *SCEL* | 4.877924 | 0.00754 |
| *AL391056.1* | 2.249982 | 0.00174 | *KLK10* | 4.901686 | 0.000108 |
| *ABHD17C* | 2.251034 | 0.003051 | *GALNT5* | 4.930599 | 0.00041 |
| *ANGPT2* | 2.252688 | 0.005083 | *KRT15* | 4.934401 | 0.001564 |
| *POC1A* | 2.260383 | 0.006494 | *S100P* | 4.938519 | 0.00657 |
| *AC245060.2* | 2.265404 | 0.002959 | *CP* | 4.989562 | 0.000286 |
| *AP000695.2* | 2.267655 | 0.002552 | *FOXL1* | 5.089792 | 0.000305 |
| *LINC01615* | 2.273374 | 0.005891 | *KLK6* | 5.110757 | 0.000305 |
| *TOX3* | 2.276285 | 0.001439 | *NMU* | 5.154631 | 0.000288 |
| *FBXL19-AS1* | 2.277956 | 0.001941 | *ADGRF1* | 5.155028 | 0.002572 |
| *FOXP3* | 2.279676 | 0.009897 | *HJURP* | 5.159304 | 0.000258 |
| *FANCB* | 2.284001 | 0.003164 | *AFAP1-AS1* | 5.169616 | 0.000899 |
| *MSL3P1* | 2.28416 | 0.00693 | *GABRP* | 5.239352 | 0.005186 |
| *ZWINT* | 2.285235 | 0.000376 | *IQGAP3* | 5.30326 | 0.000196 |
| *ADAM9* | 2.286732 | 0.000258 | *TNS4* | 5.304692 | 0.006505 |
| *PBK* | 2.287854 | 0.007028 | *IGFL2* | 5.304925 | 0.000209 |
| *PRSS22* | 2.292723 | 0.005025 | *ASPM* | 5.320061 | 0.000266 |
| *PPP1R1C* | 2.297265 | 0.00054 | *KRT16* | 5.334964 | 0.001356 |
| *MND1* | 2.304439 | 0.008189 | *UCA1* | 5.408704 | 0.000736 |
| *AP000695.1* | 2.306853 | 0.007544 | *COL17A1* | 5.462371 | 0.008285 |
| *ITGA3* | 2.314688 | 0.008312 | *LINC01559* | 5.488295 | 0.001889 |
| *C8orf31* | 2.317189 | 0.006127 | *ANLN* | 5.552304 | 0.000161 |
| *LINC01711* | 2.32398 | 0.003859 | *TMPRSS4* | 5.585764 | 0.004525 |
| *CDT1* | 2.327884 | 0.003494 | *KLK7* | 5.644152 | 0.001371 |
| *ACSL5* | 2.330282 | 0.004736 | *GJB3* | 5.69696 | 0.000209 |
| *HIST2H2BE* | 2.332792 | 0.003495 | *SFN* | 5.771243 | 1.12E-05 |
| *IL4I1* | 2.346713 | 0.009578 | *PITX1* | 5.960714 | 8.78E-05 |
| *FSCN1* | 2.348062 | 0.002347 | *MUC16* | 6.202631 | 0.003994 |
| *HIST1H2BC* | 2.348148 | 0.003807 | *TNNT1* | 6.208199 | 7.65E-05 |
| *SGPP2* | 2.350409 | 0.000907 | *PADI1* | 6.448211 | 0.002998 |

Supplementary Table 5. The top 500 DFS-related differentially expressed genes of TCGA PAAD database.

| **Gene symbol (1-250)** | **P value** | **Gene symbol (251-500)** | **P value** |
| --- | --- | --- | --- |
| *NAIP* | 4.88E-07 | *FAM183B* | 6.81E-04 |
| *RP11-54H7.4* | 1.30E-06 | *CCDC154* | 6.85E-04 |
| *NSUN6* | 4.53E-06 | *ANO10* | 6.98E-04 |
| *ZNF775* | 5.76E-06 | *RP11-304L19.2* | 6.99E-04 |
| *RP11-2B6.2* | 1.37E-05 | *RP11-250H24.2* | 7.00E-04 |
| *HIST1H1B* | 1.46E-05 | *MPZ* | 7.10E-04 |
| *RP11-258C19.7* | 1.52E-05 | *RP11-69E11.4* | 7.13E-04 |
| *LINC00641* | 1.77E-05 | *RP11-488C13.6* | 7.28E-04 |
| *AQP4-AS1* | 1.96E-05 | *AC018766.4* | 7.33E-04 |
| *COL9A2* | 2.46E-05 | *RP11-445N20.3* | 7.36E-04 |
| *RP11-111K18.2* | 2.47E-05 | *RP11-227G15.10* | 7.43E-04 |
| *CH17-340M24.3* | 2.52E-05 | *ERCC6L* | 7.43E-04 |
| *RP11-130F10.1* | 2.68E-05 | *CLEC4F* | 7.46E-04 |
| *S100A16* | 2.68E-05 | *SPDL1* | 7.55E-04 |
| *RP11-16N11.2* | 2.75E-05 | *RP3-414A15.11* | 7.55E-04 |
| *RP11-710F7.3* | 2.79E-05 | *RP11-353N14.2* | 7.56E-04 |
| *MMP28* | 2.85E-05 | *A2ML1* | 7.66E-04 |
| *RP11-269C23.5* | 2.87E-05 | *RP11-452H21.4* | 7.67E-04 |
| *SPRN* | 2.89E-05 | *FAM83H* | 7.80E-04 |
| *FKBP1A* | 3.16E-05 | *RP11-455J20.3* | 7.81E-04 |
| *BCL2L1* | 3.25E-05 | *ZNF75D* | 7.84E-04 |
| *DUSP14* | 3.36E-05 | *B4GALT1* | 7.85E-04 |
| *CTD-3222D19.12* | 3.80E-05 | *LA16c-312E8.4* | 7.87E-04 |
| *FAM83A* | 3.87E-05 | *TRIM16* | 7.88E-04 |
| *CELF6* | 4.34E-05 | *CH17-360D5.2* | 7.90E-04 |
| *RP3-525N10.2* | 4.67E-05 | *RP3-486I3.7* | 7.92E-04 |
| *JAG1* | 4.89E-05 | *IPO5P1* | 7.92E-04 |
| *HCRT* | 5.00E-05 | *TPX2* | 8.03E-04 |
| *HIGD2A* | 5.01E-05 | *GMPS* | 8.07E-04 |
| *NPIPA5* | 5.53E-05 | *ETFDH* | 8.11E-04 |
| *SMCR5* | 5.61E-05 | *AHI1* | 8.11E-04 |
| *RP4-583P15.10* | 5.75E-05 | *FSCN1* | 8.18E-04 |
| *ATP6V0A1* | 7.16E-05 | *RP11-90P13.1* | 8.21E-04 |
| *ZFHX2* | 7.22E-05 | *AL136419.6* | 8.21E-04 |
| *NEURL4* | 7.29E-05 | *RP11-620J15.3* | 8.23E-04 |
| *WAC-AS1* | 7.30E-05 | *RP11-170N16.3* | 8.24E-04 |
| *RP11-295H24.3* | 7.56E-05 | *CTD-2081C10.7* | 8.27E-04 |
| *ZBTB48* | 7.64E-05 | *FAR2P1* | 8.30E-04 |
| *RAPGEF4* | 7.85E-05 | *RP11-524O1.4* | 8.36E-04 |
| *SCAMP5* | 7.89E-05 | *S100A10* | 8.48E-04 |
| *RP11-708J19.1* | 7.89E-05 | *TGM2* | 8.51E-04 |
| *NPIPA7* | 8.29E-05 | *TECTA* | 8.59E-04 |
| *PSMA2* | 8.60E-05 | *KIF18A* | 8.61E-04 |
| *PTGES* | 8.71E-05 | *ZNF185* | 8.67E-04 |
| *HERC1* | 8.74E-05 | *SEC14L2* | 8.78E-04 |
| *LINC00930* | 8.82E-05 | *KCNC3* | 8.82E-04 |
| *SYNGR1* | 9.00E-05 | *RP11-468E2.5* | 8.85E-04 |
| *RP11-686O6.2* | 9.02E-05 | *SH3GL1P3* | 8.86E-04 |
| *BZRAP1* | 9.13E-05 | *RP11-89K11.1* | 8.93E-04 |
| *BRINP2* | 9.38E-05 | *MAOB* | 9.01E-04 |
| *ENAM* | 9.39E-05 | *PPP1R1A* | 9.04E-04 |
| *RP11-285E9.6* | 9.72E-05 | *AGFG2* | 9.17E-04 |
| *ANO1* | 1.04E-04 | *RP11-817O13.9* | 9.19E-04 |
| *CTD-3080P12.3* | 1.06E-04 | *RP11-304L19.4* | 9.20E-04 |
| *GPN1* | 1.09E-04 | *CCDC130* | 9.24E-04 |
| *CTD-3193K9.11* | 1.09E-04 | *KLK9* | 9.33E-04 |
| *NKX3-1* | 1.12E-04 | *TOP2A* | 9.34E-04 |
| *RP11-787I22.3* | 1.12E-04 | *RP11-74E22.3* | 9.39E-04 |
| *CTB-176F20.3* | 1.15E-04 | *EIF6* | 9.40E-04 |
| *CTD-2293H3.1* | 1.19E-04 | *RP11-358N2.2* | 9.40E-04 |
| *RP11-1105G2.3* | 1.19E-04 | *LINC01314* | 9.43E-04 |
| *ZNF488* | 1.19E-04 | *RP11-459F6.3* | 9.43E-04 |
| *IL11RA* | 1.21E-04 | *EFR3B* | 9.46E-04 |
| *RP11-714G18.1* | 1.28E-04 | *IRAK2* | 9.49E-04 |
| *RP11-488C13.5* | 1.29E-04 | *PRIMPOL* | 9.53E-04 |
| *KIAA1683* | 1.32E-04 | *RP11-278C7.4* | 9.60E-04 |
| *PCP4* | 1.35E-04 | *EIF4EBP1* | 9.64E-04 |
| *ADRB1* | 1.37E-04 | *RP11-76C10.5* | 9.73E-04 |
| *CACNA1C-AS1* | 1.38E-04 | *RP11-629G13.1* | 9.74E-04 |
| *RP11-402D21.2* | 1.40E-04 | *CTB-50L17.16* | 9.78E-04 |
| *SPTBN2* | 1.42E-04 | *TMEM205* | 9.81E-04 |
| *PLA2G16* | 1.44E-04 | *KB-1125A3.11* | 9.83E-04 |
| *ARNTL2* | 1.45E-04 | *RP11-182J1.3* | 9.87E-04 |
| *LY6D* | 1.49E-04 | *DEPDC1* | 9.91E-04 |
| *CTSV* | 1.49E-04 | *CDH3* | 9.91E-04 |
| *FLJ38576* | 1.52E-04 | *RP11-478J18.2* | 9.94E-04 |
| *MANSC4* | 1.56E-04 | *RP11-762L8.6* | 1.00E-03 |
| *RP11-355O1.11* | 1.57E-04 | *CLCN3P1* | 1.01E-03 |
| *C16orf74* | 1.59E-04 | *MBLAC1* | 1.01E-03 |
| *RP11-422P24.11* | 1.60E-04 | *CDIP1* | 1.01E-03 |
| *RP1-100J12.1* | 1.60E-04 | *TUSC8* | 1.03E-03 |
| *CHRNA10* | 1.62E-04 | *MRPL11* | 1.04E-03 |
| *F3* | 1.62E-04 | *YBX3* | 1.04E-03 |
| *INSIG2* | 1.64E-04 | *AP001062.7* | 1.05E-03 |
| *NFE2L3* | 1.65E-04 | *ALPP* | 1.05E-03 |
| *RP11-127B20.3* | 1.66E-04 | *TRIM52* | 1.06E-03 |
| *CTC-524C5.2* | 1.67E-04 | *PAH* | 1.06E-03 |
| *RP11-267M23.4* | 1.68E-04 | *CTD-3001H11.2* | 1.08E-03 |
| *LINC01605* | 1.68E-04 | *MDFI* | 1.08E-03 |
| *TAT-AS1* | 1.72E-04 | *GTSE1* | 1.08E-03 |
| *ITGA3* | 1.72E-04 | *RP11-386M24.6* | 1.09E-03 |
| *MAGEH1* | 1.76E-04 | *RP11-347I19.8* | 1.10E-03 |
| *PGK1P2* | 1.76E-04 | *RP11-989E6.13* | 1.10E-03 |
| *FAM228B* | 1.82E-04 | *AC005534.6* | 1.11E-03 |
| *CTD-3131K8.2* | 1.84E-04 | *CENPI* | 1.11E-03 |
| *PHKA2* | 1.91E-04 | *UBXN2A* | 1.11E-03 |
| *LINC00869* | 1.91E-04 | *AGAP6* | 1.12E-03 |
| *CTC-529P8.1* | 1.97E-04 | *KRT7* | 1.12E-03 |
| *TEAD4* | 2.00E-04 | *BZW1* | 1.12E-03 |
| *STAM-AS1* | 2.02E-04 | *PPIAL4G* | 1.13E-03 |
| *KARS* | 2.15E-04 | *NSFL1C* | 1.14E-03 |
| *RBM22P2* | 2.21E-04 | *RP11-230C9.4* | 1.14E-03 |
| *KIAA0408* | 2.24E-04 | *RP11-360L9.4* | 1.14E-03 |
| *FFAR3* | 2.26E-04 | *PPP1R3E* | 1.18E-03 |
| *B4GALT5* | 2.28E-04 | *RND2* | 1.18E-03 |
| *RP1-278C19.8* | 2.32E-04 | *AXL* | 1.19E-03 |
| *PRR26* | 2.37E-04 | *FAM86C1* | 1.20E-03 |
| *ALOX12-AS1* | 2.38E-04 | *TMEM41A* | 1.20E-03 |
| *ADAM10* | 2.40E-04 | *CTD-2369P2.10* | 1.21E-03 |
| *ADAM9* | 2.41E-04 | *ANXA2P1* | 1.21E-03 |
| *U2AF1L4* | 2.45E-04 | *MEST* | 1.21E-03 |
| *ZBED2* | 2.49E-04 | *GNG7* | 1.21E-03 |
| *KLK6* | 2.51E-04 | *SUSD2* | 1.21E-03 |
| *KLHL41* | 2.53E-04 | *SGMS1-AS1* | 1.22E-03 |
| *TMEM43* | 2.53E-04 | *ZNF540* | 1.24E-03 |
| *PDCL3P4* | 2.53E-04 | *A1CF* | 1.24E-03 |
| *RP11-484K9.4* | 2.54E-04 | *RP4-791M13.3* | 1.24E-03 |
| *RP11-182J1.1* | 2.57E-04 | *LLNLR-268E12.1* | 1.25E-03 |
| *SAT2* | 2.57E-04 | *RP11-106M3.2* | 1.26E-03 |
| *C19orf44* | 2.59E-04 | *KLK10* | 1.26E-03 |
| *DIEXF* | 2.63E-04 | *ST20-AS1* | 1.26E-03 |
| *C6orf15* | 2.71E-04 | *KIAA1524* | 1.27E-03 |
| *TEX22* | 2.73E-04 | *RP11-338N10.1* | 1.27E-03 |
| *SLC25A14* | 2.74E-04 | *NFASC* | 1.29E-03 |
| *FAM95B1* | 2.78E-04 | *PNPLA7* | 1.29E-03 |
| *RP11-327P2.5* | 2.78E-04 | *KDM8* | 1.30E-03 |
| *RASA4CP* | 2.78E-04 | *RP11-134G8.5* | 1.30E-03 |
| *RP11-53M11.3* | 2.87E-04 | *RP11-673E1.1* | 1.30E-03 |
| *PTPRS* | 2.89E-04 | *RP11-3D4.3* | 1.31E-03 |
| *RP4-758J18.2* | 2.90E-04 | *CTB-60B18.15* | 1.31E-03 |
| *AC142472.6* | 2.93E-04 | *CAPN10-AS1* | 1.31E-03 |
| *SSPO* | 2.99E-04 | *CD274* | 1.31E-03 |
| *LRRC37B* | 3.02E-04 | *CTC-359D24.3* | 1.32E-03 |
| *AC005154.7* | 3.05E-04 | *WDR43* | 1.32E-03 |
| *SLC9B1* | 3.08E-04 | *CASC8* | 1.32E-03 |
| *SERPINE1* | 3.10E-04 | *PCDH1* | 1.33E-03 |
| *NAB1* | 3.13E-04 | *ACER1* | 1.33E-03 |
| *RP11-305L7.1* | 3.14E-04 | *CTD-2623N2.3* | 1.34E-03 |
| *NUTM2HP* | 3.14E-04 | *LRRC59* | 1.34E-03 |
| *CBX7* | 3.17E-04 | *OSGEP* | 1.35E-03 |
| *NDUFB8P2* | 3.18E-04 | *C19orf71* | 1.35E-03 |
| *ZNF436-AS1* | 3.20E-04 | *RPE* | 1.36E-03 |
| *AC098820.4* | 3.24E-04 | *YKT6* | 1.36E-03 |
| *CTB-31O20.2* | 3.27E-04 | *ARL4C* | 1.36E-03 |
| *RPARP-AS1* | 3.27E-04 | *RP11-672L10.6* | 1.36E-03 |
| *NYAP1* | 3.31E-04 | *SMYD5* | 1.36E-03 |
| *WNK2* | 3.32E-04 | *LINC00973* | 1.37E-03 |
| *PI4KAP1* | 3.33E-04 | *CTD-2619J13.23* | 1.37E-03 |
| *AC005224.2* | 3.34E-04 | *RP11-37C7.3* | 1.38E-03 |
| *ANLN* | 3.36E-04 | *PDZD4* | 1.38E-03 |
| *DCBLD2* | 3.38E-04 | *HNRNPA1P33* | 1.38E-03 |
| *ZNF18* | 3.38E-04 | *PLCXD2* | 1.38E-03 |
| *RP11-178H8.7* | 3.43E-04 | *CNBP* | 1.39E-03 |
| *BIRC5* | 3.53E-04 | *EPHX4* | 1.40E-03 |
| *LA16c-306A4.2* | 3.59E-04 | *TTC6* | 1.40E-03 |
| *COLQ* | 3.62E-04 | *ZDHHC11B* | 1.40E-03 |
| *AC006547.14* | 3.69E-04 | *DKK1* | 1.42E-03 |
| *NLRP1* | 3.70E-04 | *RAC1* | 1.44E-03 |
| *MTERF2* | 3.74E-04 | *CCDC7* | 1.47E-03 |
| *MT2A* | 3.75E-04 | *CCER2* | 1.47E-03 |
| *ZNF467* | 3.76E-04 | *RP11-445N20.2* | 1.48E-03 |
| *GSTM2* | 3.77E-04 | *C1orf204* | 1.48E-03 |
| *MAN1C1* | 3.77E-04 | *COLGALT1* | 1.48E-03 |
| *RRM2* | 3.79E-04 | *RP11-380L11.4* | 1.48E-03 |
| *RP4-713A8.1* | 3.79E-04 | *BEX4* | 1.49E-03 |
| *RP11-61J19.5* | 3.83E-04 | *CTD-2537I9.18* | 1.50E-03 |
| *SCAND2P* | 3.85E-04 | *GFM1* | 1.50E-03 |
| *ACACB* | 3.87E-04 | *UNC119* | 1.51E-03 |
| *KIF23* | 3.91E-04 | *PCSK2* | 1.51E-03 |
| *MCOLN1* | 3.92E-04 | *KIF15* | 1.51E-03 |
| *RP11-347C18.3* | 4.00E-04 | *CDK2AP1* | 1.51E-03 |
| *FAM69B* | 4.02E-04 | *HEBP1* | 1.52E-03 |
| *NPIPA3* | 4.03E-04 | *RAD51* | 1.53E-03 |
| *BRINP1* | 4.04E-04 | *RP11-793H13.11* | 1.53E-03 |
| *RFT1* | 4.06E-04 | *CTD-2506P8.6* | 1.54E-03 |
| *DMXL2* | 4.09E-04 | *SAMD9* | 1.54E-03 |
| *ZNRD1-AS1* | 4.11E-04 | *PPIA* | 1.55E-03 |
| *CXXC4* | 4.14E-04 | *GINS1* | 1.55E-03 |
| *RP11-766N7.3* | 4.17E-04 | *DSCAML1* | 1.55E-03 |
| *FAM186A* | 4.18E-04 | *CKS1B* | 1.55E-03 |
| *RP11-281O15.4* | 4.21E-04 | *CYP2D6* | 1.56E-03 |
| *PHKA2-AS1* | 4.24E-04 | *MCM8* | 1.57E-03 |
| *SOCS2-AS1* | 4.30E-04 | *FLJ26850* | 1.57E-03 |
| *U47924.32* | 4.39E-04 | *CTD-2587M2.1* | 1.58E-03 |
| *NT5E* | 4.40E-04 | *AC005517.3* | 1.58E-03 |
| *RHD* | 4.46E-04 | *RP11-981P6.1* | 1.58E-03 |
| *MET* | 4.48E-04 | *DLGAP5* | 1.59E-03 |
| *SLC26A11* | 4.49E-04 | *RP5-1112D6.8* | 1.59E-03 |
| *GKAP1* | 4.54E-04 | *HIPK2* | 1.59E-03 |
| *GNAZ* | 4.57E-04 | *CGB7* | 1.60E-03 |
| *AP001412.1* | 4.59E-04 | *SOBP* | 1.60E-03 |
| *PIPOX* | 4.59E-04 | *RP11-196G11.2* | 1.60E-03 |
| *SERPINB5* | 4.60E-04 | *CTD-2293H3.2* | 1.60E-03 |
| *PERP* | 4.63E-04 | *ZGLP1* | 1.60E-03 |
| *PLA2G4C* | 4.65E-04 | *RP11-411B10.7* | 1.61E-03 |
| *RP11-83A24.2* | 4.70E-04 | *CAMTA2* | 1.61E-03 |
| *CORT* | 4.73E-04 | *SPTLC1* | 1.61E-03 |
| *CLN3* | 4.73E-04 | *RP11-441O15.3* | 1.61E-03 |
| *GALT* | 4.82E-04 | *TGM4* | 1.61E-03 |
| *FAM101A* | 4.83E-04 | *CTB-119C2.1* | 1.61E-03 |
| *HMGB3P24* | 4.94E-04 | *HRH3* | 1.62E-03 |
| *PNO1* | 4.98E-04 | *CENPL* | 1.63E-03 |
| *KIF2C* | 4.99E-04 | *RP3-329A5.8* | 1.64E-03 |
| *PRKG1-AS1* | 5.04E-04 | *C1orf132* | 1.65E-03 |
| *TBC1D2* | 5.06E-04 | *RP11-467D6.1* | 1.66E-03 |
| *ITGB5* | 5.07E-04 | *CRYBA4* | 1.66E-03 |
| *ANKRD18B* | 5.11E-04 | *TICRR* | 1.66E-03 |
| *C14orf93* | 5.26E-04 | *RP11-503C24.6* | 1.67E-03 |
| *AC018647.3* | 5.29E-04 | *CHST12* | 1.67E-03 |
| *RP1-265C24.8* | 5.35E-04 | *DTL* | 1.68E-03 |
| *ACRBP* | 5.45E-04 | *SALL2* | 1.69E-03 |
| *TMOD3* | 5.46E-04 | *NDUFA6-AS1* | 1.71E-03 |
| *CCDC28B* | 5.50E-04 | *RP11-1109F11.5* | 1.72E-03 |
| *RP11-395I6.3* | 5.52E-04 | *AC112721.1* | 1.72E-03 |
| *ZMAT1* | 5.53E-04 | *NLN* | 1.72E-03 |
| *POU6F1* | 5.56E-04 | *GUSB* | 1.74E-03 |
| *ANKRD19P* | 5.57E-04 | *NRIP2* | 1.74E-03 |
| *CYP2C8* | 5.59E-04 | *C9orf147* | 1.75E-03 |
| *LINC01481* | 5.60E-04 | *IFT88* | 1.75E-03 |
| *CTD-2587H24.10* | 5.61E-04 | *TYK2* | 1.76E-03 |
| *LINC00152* | 5.61E-04 | *TRIB2* | 1.76E-03 |
| *VANGL1* | 5.69E-04 | *AJ003147.11* | 1.76E-03 |
| *ANXA2* | 5.73E-04 | *RP11-434B12.1* | 1.77E-03 |
| *NAA50* | 5.77E-04 | *FERMT1* | 1.78E-03 |
| *SLC25A35* | 5.78E-04 | *LINC00957* | 1.78E-03 |
| *RP11-274B21.9* | 5.79E-04 | *CELF4* | 1.78E-03 |
| *RP11-407N17.5* | 5.84E-04 | *RP11-513I15.6* | 1.79E-03 |
| *RP11-261P13.6* | 5.85E-04 | *ASAP2* | 1.79E-03 |
| *RNF121* | 5.87E-04 | *PNMA3* | 1.80E-03 |
| *KCTD4* | 5.88E-04 | *CCDC108* | 1.80E-03 |
| *FAM25A* | 5.89E-04 | *RP11-644F5.11* | 1.80E-03 |
| *RP11-635N19.1* | 5.93E-04 | *AGBL3* | 1.81E-03 |
| *EFNB2* | 5.93E-04 | *SKIL* | 1.82E-03 |
| *ARHGAP23* | 5.94E-04 | *TMEM200B* | 1.82E-03 |
| *RP11-188P17.2* | 5.98E-04 | *ARMC10* | 1.82E-03 |
| *SHCBP1* | 5.99E-04 | *COMMD2* | 1.83E-03 |
| *LDHA* | 6.10E-04 | *RP11-169K17.3* | 1.83E-03 |
| *AC005538.3* | 6.19E-04 | *LINC01016* | 1.84E-03 |
| *LRRC16B* | 6.31E-04 | *RP11-803D5.1* | 1.85E-03 |
| *CXorf57* | 6.32E-04 | *RP5-1085F17.3* | 1.85E-03 |
| *EML6* | 6.57E-04 | *MAP7D2* | 1.86E-03 |
| *ALPPL2* | 6.57E-04 | *LEAP2* | 1.88E-03 |
| *RP13-616I3.1* | 6.59E-04 | *TUBA3FP* | 1.88E-03 |
| *RSAD1* | 6.64E-04 | *CRYM* | 1.88E-03 |
| *WNT7A* | 6.67E-04 | *DYNC1I1* | 1.89E-03 |
| *RP11-131L12.4* | 6.68E-04 | *NDC1* | 1.89E-03 |
| *KCTD19* | 6.71E-04 | *LRRD1* | 1.89E-03 |
| *PRR22* | 6.73E-04 | *ZDHHC3* | 1.90E-03 |
| *XXbac-BPG299F13.17* | 6.78E-04 | *MMRN1* | 1.90E-03 |
| *CIRBP* | 6.81E-04 | *LINC00900* | 1.93E-03 |
